# Long-term immunoprophylaxis by TLR9 ligand mediates macrophage-ILC cross-talk against respiratory viral infection

**DOI:** 10.1126/sciadv.aeh6480

**Published:** 2026-10-09

**Authors:** Asuka Joy Tobuse, Kouji Kobiyama, Jun Tsuchida, Masamitsu N. Asaka, Daichi Utsumi, Mariana Silva Almeida, Yaeko Nakajima-Takagi, Motohiko Oshima, Tomoya Hayashi, Burcu Temizoz, Etsushi Kuroda, Cevayir Coban, Yasuhiro Yasutomi, Atsushi Iwama, Ken J. Ishii

**Affiliations:** ^1^ Division of Vaccine Science, Department of Microbiology and Immunology, The Institute of Medical Science, The University of Tokyo, Tokyo, Japan.; ^2^ Department of Computational Biology and Medical Science, Graduate School of Frontier Sciences, The University of Tokyo, Tokyo, Japan.; ^3^ International Vaccine Design Center, The Institute of Medical Science, The University of Tokyo, Tokyo, Japan.; ^4^ Division of Rheumatology, Department of Medicine, University of California San Diego, La Jolla, CA, USA.; ^5^ Laboratory of Immunoregulation and Vaccine Research, Tsukuba Primate Research Center, National Institutes of Biomedical Innovation, Health and Nutrition, Tsukuba, Japan.; ^6^ Leprosy Research Center, National Institute of Infectious Diseases, Japan Institute for Health Security, Tokyo, Japan.; ^7^ Division of Malaria Immunology, Department of Microbiology and Immunology, The Institute of Medical Science, The University of Tokyo, Tokyo, Japan.; ^8^ The University of Tokyo Pandemic Preparedness, Infection and Advanced Research Center (UTOPIA), University of Tokyo, Tokyo, Japan.; ^9^ Division of Stem Cell and Molecular Medicine, Center for Stem Cell Biology and Regenerative Medicine, The Institute of Medical Science, University of Tokyo, Tokyo, Japan.; ^10^ Department of Immunology, Hyogo Medical University School of Medicine, Nishinomiya, Hyogo, Japan.; ^11^ Institute for Vaccine Research and Development, Hokkaido University, Sapporo, Hokkaido, Japan.

## Abstract

Rapid development of host immunity is critical during the emergence of viral outbreaks when vaccines are not yet available. Here, we demonstrate that CpG oligodeoxynucleotide-schizophyllan complex (K3-SPG) induces antigen-independent, long-term protection against lethal respiratory virus infection. A single intranasal administration successfully enhanced host resistance to infection for as long as 100 days posttreatment. Mechanistically, we observed a lung-restricted biphasic innate activation of macrophages and innate lymphoid cells. Early protection was mediated by CD11b^+^CD11c^+^ macrophages (DP MΦ) and monocyte-derived interstitial macrophages (IMΦ). Late protection was mediated by innate lymphoid cells that were epigenetically reprogrammed, suggesting that two distinct innate immune subsets contribute to the maintenance of disease tolerance in the lung microenvironment. Deficiency of TLR9 or TNF-α abrogated innate activation and protective immunity. Collectively, these findings demonstrate that adjuvant-induced prophylaxis is a promising approach to enhancing host resistance against respiratory virus infection, involving the biphasic cooperation between innate immune cells in the local tissue.

## INTRODUCTION

The past few decades have reminded us of the devastating impact pandemics and outbreaks can have on global health and society. As most recently seen in the severe acute respiratory syndrome coronavirus 2 (SARS-CoV-2) pandemic from 2019, although several types of vaccines were introduced at a remarkable pace, millions of infection cases and 1.78 million deaths were reported by 18 December 2020 (*1*). Moreover, the current spread of highly pathogenic avian influenza strains which affected millions of livestock globally has raised concerns of the spill over into humans which may lead to human-to-human transmission (*2*). Because of the gap in vaccine coverage, in 2025 alone, the number of measles cases in Canada showed an approximate 30-fold increase from 2024 (*3*). While the occurrence of the next pandemic is unwanted, the possibility cannot be eliminated.

The gap between pathogen emergence and availability of vaccine and therapies remains a hurdle in global health care. To respond to global efforts in pandemic preparedness, the Coalition for Epidemic Preparedness Innovations has announced a “moonshot” goal of vaccine development and distribution within 100 days of the emergence of a new pathogen, and this goal has been adopted by governments in various countries including Japan (*4*). In addition to vaccines and therapeutics, emergency response remains a challenge due to the inability to predict the next pandemic pathogen and the lack of information on newly emergent pathogens. To prepare for the next pandemic, it is imperative to develop alternative strategies that can be stockpiled and induce broad protection.

The concept of nonspecific immunoprophylaxis dates back to the 1920s (*5*, *6*), when the Bacille Calmette-Guérin (BCG) vaccine, originally intended for tuberculosis, was reported to confer nonspecific protection against unrelated pathogens (*7*). Despite its efficacy, global distribution of live BCG vaccination as an emergency measure in the next pandemic is not feasible due to safety concerns, primarily in individuals with genetic deficiencies and communities where HIV is prevalent (*8*, *9*). To overcome this limitation, global efforts are ongoing in search for an appropriate innate memory inducer which can provide temporary protection against unknown pathogens, such as adjuvants, while vaccines and antigen-specific therapies are under development.

Adjuvants are known to activate innate immune responses and enhance vaccine efficacy. Depending on the type of adjuvant, there are several molecular targets identified, including Toll-like receptors (TLRs), cytosolic pattern recognition receptors such as RIG-I and STING, and C-type lectin receptors such as Dectin-1 and Mincle (*10*–*12*). In addition, reports show that adjuvants can confer protection against infections without pathogen-derived antigens, via modulation of epigenetic and metabolic pathways, and induction of cell death–related processes for innate induction (*10*). Abundant studies confirmed the immunoprophylactic effect of adjuvants in the 1990s (*13*–*16*), and most recently, immunostimulatory compounds such as TLR ligands and β-glucan polysaccharides became key players for their capacity to confer nonspecific protection and innate immune memory (*17*, *18*). This phenomenon of adjuvant-induced memory is also commonly referred to as trained immunity, which defines the innate immune memory that induces enhanced innate immune activation and cytokine production in “trained” innate immune cells. Recent studies elucidated that trained immunity can be sustained by reprogramming of cellular metabolism, epigenome, or by the reprogramming of the hematopoietic stem cell compartment (*17*, *18*). Simultaneously, few studies have shed light on the regulatory roles of inflammatory cytokines, such as the cooperation of tumor necrosis factor–α (TNF-α) and type I interferons (IFNs) in reprogramming the macrophage epigenome (*19*). Another mechanism underlying adjuvant-mediated protection is the enhancement of disease tolerance (*20*). Here, the priming and reprogramming of innate immune cells shape their response and contribute to improved tissue integrity during acute infection without affecting viral replication. While there are still numerous unanswered questions to the understanding of adjuvant-mediated immunoprophylaxis, it is now known that we can leverage the innate stimulatory properties of adjuvants for various clinical applications, including prophylaxis against infections.

Here, we aim to refine the mechanism of innate immune memory against respiratory viruses by using CpG oligodeoxynucleotide (ODN). CpG ODNs are synthetic, short single-stranded DNA molecules containing unmethylated CpG motif(s) and are known to be potent inducers of innate immune responses through TLR9 (*20**–**22*). In a previous review, Ishii *et al.* (*15*) reported on the capability of CpG ODNs alone to confer 100% survival in mouse models of infection against *Listeria monocytogenes*, *Leishmania major*, *Mycobacterium tuberculosis*, and prion infections. However, its efficacy against viral infections remains insufficient. On the other hand, β-glucan induces innate activation via Dectin-1 and has become a commonly used innate immune memory inducer against several infection models, including tuberculosis (*23*) and influenza (*24*). Notably, β-glucan–induced innate immune memory has been demonstrated in both murine models and human monocytes, suggesting that this phenomenon may be conserved across species (*23*). However, one challenge is the dose and route of administration of β-glucan required to induce training of myeloid cells in these models. Several papers report successful training by administering as much as 1 mg of β-glucan per mouse by intraperitoneal injection (*23**–**25*). While other routes of administration could be considered, such as oral administration, a previous pilot study reported that oral β-glucan failed to induce innate immune activation, despite a high dose of 1000 mg once daily for 7 weeks (*26*). This raises a concern on the translational aspect of standard β-glucan formulations. In 2014, we demonstrated the complexation of K3 CpG ODN with schizophyllan (SPG), a pure β-glucan, termed as “K3-SPG” (*27*). Having a unique triple helical structure, its nanosized property enables efficient uptake by immune cells such as macrophages and is a potent inducer of type I and II IFNs, alongside proinflammatory cytokines such as interleukin-6 (IL-6), IL-12, and TNF-α. Early studies have determined its dependency on TLR9 signaling but not Dectin-1 signaling for the adjuvant effects of K3-SPG due to the soluble state of SPG that is unable to activate Dectin-1–mediated innate immune responses (*27*). Moreover, its potent adjuvanticity with the influenza hemagglutinin split vaccine (*28*) and antigen-independent antitumor properties in an in situ vaccination study against pancreaticobiliary and gastrointestinal cancer models (*29*–*31*) were previously explored.

In this study, we explore the potential of K3-SPG to confer antigen-independent protection against a respiratory virus infection model. Our findings demonstrate that a single administration of K3-SPG (*27*) can confer nonspecific protection against respiratory viruses, characterized by early but sustained activation of lung macrophages, followed by induction of innate lymphoid cell (ILC) subsets which undergo epigenetic reprogramming for long-term protection. By leveraging on the capacity of innate immune cells, we propose the use of adjuvants as an immunoprophylactic approach to curbing mortality and infections as a feasible strategy for future pandemics.

## RESULTS

### K3-SPG mediates long-term protection against influenza A virus without antigen

To assess the immunoprophylactic potential of K3-SPG, mice were treated intranasally with K3-SPG, K3-dA_40_ that was used in complexation for K3-SPG, TLR3 agonist Poly(I:C), mouse-specific TLR9 agonist ODN1668, and β-glucans (curdlan, zymosan, zymosan depleted, and SPG). Among the β-glucans used, zymosan refers to the insoluble *Saccharomyces cerevisiae* cell wall extract, while zymosan depleted refers to zymosan depleted of TLR2 activity achieved by hot alkali treatment of zymosan (*32*). At 3 days postadministration, mice were exposed to a lethal dose of influenza A virus [A/PR/8/34 strain (PR8); 2LD_50_, 1 × 10^2^ plaque-forming units (PFU)] and monitored for survival and body weight loss for up to 20 days postinfection (dpi) (Fig. 1A). Mice pretreated with K3-SPG exhibited less severe body weight loss and high survival rate after viral infection compared to other treatments. K3-dA_40_ and ODN1668 displayed comparable efficacy to K3-SPG, although β-glucans treatment showed lower protection than the phosphate-buffered saline (PBS)–treated group. These data demonstrate the immunoprophylactic potential of TLR9 ligand against respiratory virus infection. Individual experiment datasets are provided in fig. S1.

**Fig. 1.**
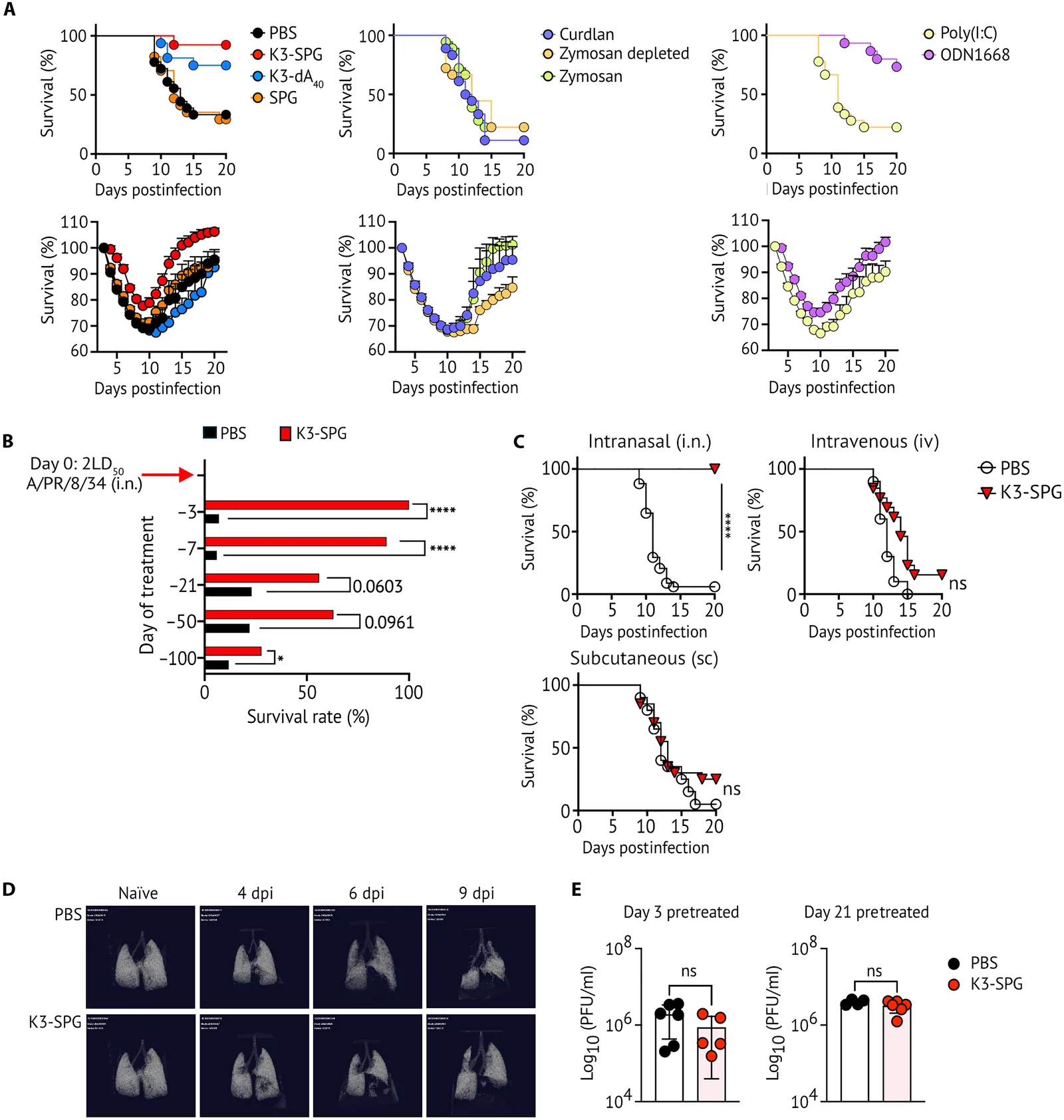
A single administration of K3-SPG mediates long-term protection against influenza A virus without antigen. (**A**) Survival and body weight changes following A/PR/8/34 (PR8, 1 × 10^2^ PFU) infection in PBS or adjuvant pretreated mice via intranasal injection. Data are pooled from three independent experiments, with *n* = 6 to 7 for each independent experiment. (**B**) Survival of mice following lethal PR8 infection in mice pretreated with PBS or K3-SPG via intranasal injection at 3, 7, 21, 50, and 100 days before infection. Survival was monitored up to 20 dpi. Data are pooled from two independent experiments (*n* = 6 per experiment) for day 21, 50, and 100. Data are pooled from three independent experiments (*n* = 6 to 8 per experiment) for days 3 and 7. (**C**) Survival curves of mice pretreated with PBS or K3-SPG via intranasal, intravenous, or subcutaneous injection, followed by lethal PR8 infection at 3 days posttreatment. Data are pooled from three independent experiments (intranasal, *n* = 10 to 12 per experiment) and from one experiment (intravenous and subcutaneous; *n* = 10). (**D**) Time-course micro-CT scans of lungs of PBS or K3-SPG pretreated mice. At 3 days after intranasal treatment, mice were infected with 2LD_50_ PR8 virus, and lung pathology was monitored by real-time CT imaging, up to the mortality of PBS-treated mice at 9 dpi. Images shown are representative of one experiment with *n* = 5 per treatment group. (**E**) Viral titer measurement by plaque assay of BALF isolated at 3 dpi from day 3 and day 21 pretreated mice. Data shown are pooled from two independent experiments, with *N* = 2 to 3 for each independent experiment. Survival comparison conducted with Kaplan-Maier log-rank (Mantel-Cox) test, *P ≤ 0.05 and ****P < 0.0001 [(B) and (C)]. ns, not significant.

Subsequently, we conducted a long-term protection study to investigate the duration of immunoprophylactic efficacy of K3-SPG. While waning of protection could be observed with time, survival of mice treated 100 days before virus exposure was statistically significantly higher compared to PBS-treated mice (Fig. 1B). To determine whether long-term protection is dependent on TLR9 agonist alone, we treated mice with SPG or K3-dA_40_ 21 days before viral challenge (fig. S2A). Neither group showed enhanced protection compared to the PBS-treated group, suggesting that this phenomenon was restricted to K3-SPG, which is composed of TLR9 ligand and β-glucan as a complex. We further investigated the protection against a higher lethal dose (10LD_50_, 5 × 10^2^ PFU) of PR8 infection. Administration of K3-SPG 7 days before infection showed protection even against a more severe infection (fig. S2B). To test with other respiratory viruses, we assessed SARS-CoV-2 infection using human angiotensin-converting enzyme 2 (hACE2) transgenic mice (fig. S2C). Similar survival rate as that of the PR8 10LD_50_ challenge was observed, while PBS-treated mice succumbed to infection by 9 dpi. These results suggest that pretreatment with K3-SPG protects mice from lethal respiratory viral infection.

We next examined different routes of administration (intranasal, subcutaneous, and intravenous via the tail vein) to investigate whether specific route of administration is important for the protection against influenza virus infection (Fig. 1C). Only intranasal K3-SPG treatment conferred the protection, while subcutaneous treatment showed partial protection, followed by intravenous injection which demonstrated lowest efficacy among the three. These findings suggest that local immune activation, especially in the lung, is critical for combating respiratory viral infections, at least in the case of influenza virus challenge model.

Consequently, we investigated whether K3-SPG protects mice via antiviral mechanism or modulation of host immunity. Expectedly, using real-time micro-CT, we observed less severe lung pathology in mice pretreated with K3-SPG, with recovery to infection-induced edema observed from 9 dpi (Fig. 1D). However, when we measured viral titer in bronchoalveolar lavage fluid (BALF) collected at 3 dpi, we could not observe reduced viral titers in mice pretreated with K3-SPG at day 3 or day 21 (Fig. 1E). These data suggest that K3-SPG primarily acts on host cells to enhance immunity, rather than on the virus to inhibit replication.

### Robust induction of macrophages in the lungs at single-cell resolution

To elucidate the underlying mechanism of K3-SPG, we performed single-cell RNA sequencing (scRNA-seq) on whole lung tissue at 1 and 3 days posttreatment of K3-SPG administered intranasally. TLR9 expression is largely confined to immune cells such as B cells and dendritic cells (DCs). Therefore, we excluded nonimmune cells from further analysis to improve the resolution of the target clusters. To better distinguish innate and adaptive immune populations, we separately analyzed the adaptive (T and B cell clusters; fig. S3A) and the innate compartment (Fig. 2A).

**Fig. 2.**
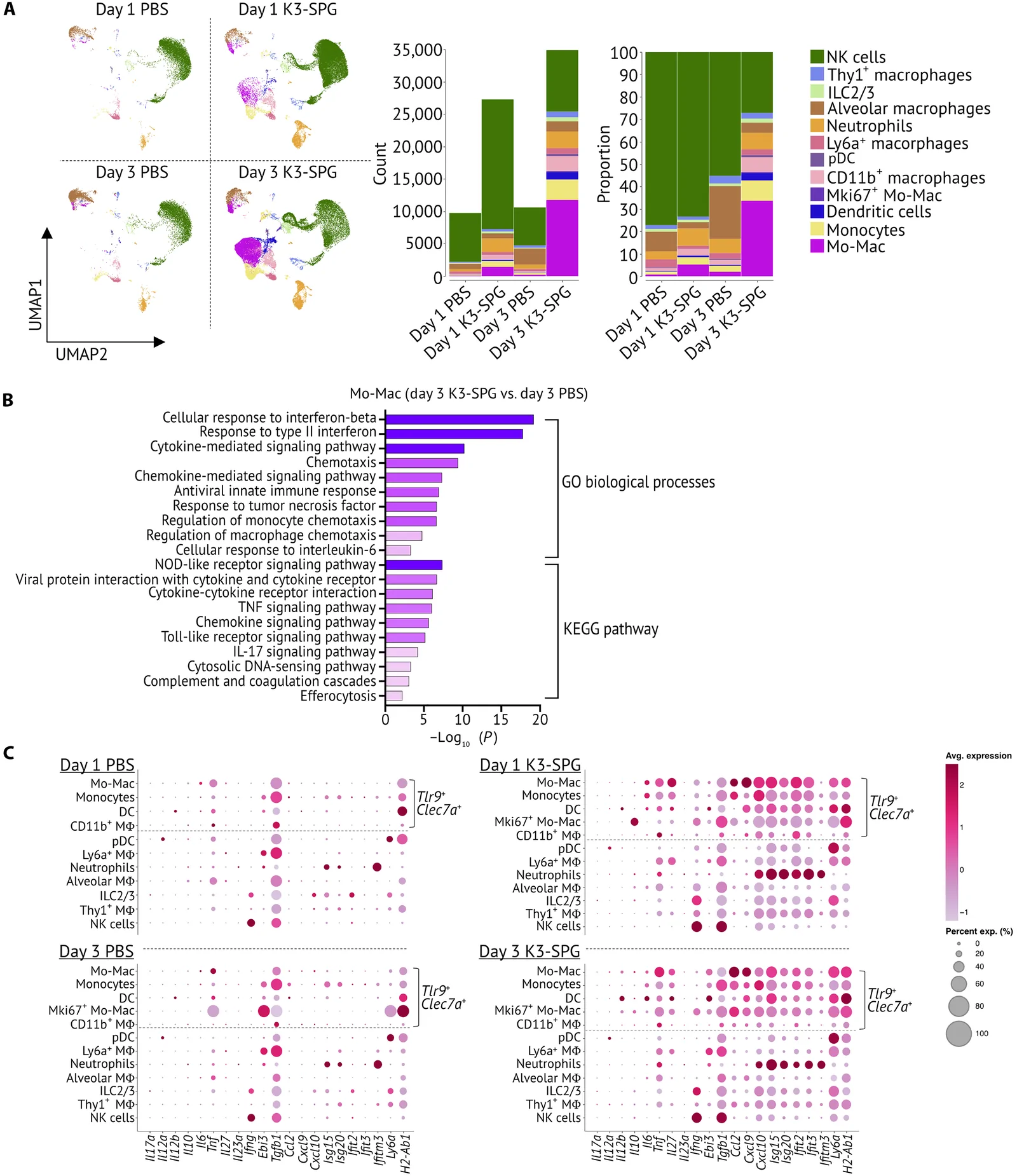
Immune cell profiling at the single-cell level reveals induction of rare myeloid cells. (**A**) scRNA-seq via the 10X Genomics platform was performed with 1.6 × 10^4^ lung cells after exclusion of dead cells from day 1 and day 3 pretreated mice (*n* = 3). Left displays the UMAP representing myeloid cell compartment after exclusion of T cells, B cells, and nonimmune cells. Right displays the bar graph presenting the change in proportions of subsets across time and treatment. One independent experiment was conducted (*n* = 3). (**B**) Bar graph demonstrates the top 10 enriched KEGG pathways and GO biological processes, respectively, in day 3 K3-SPG versus day 3 PBS for Mo-Mac cluster. Analysis was performed with Metascape. (**C**) Dot plots demonstrating the activation of subsets in order of *Tlr9*/*Clec7a* coexpression. Dotted line separates the clusters which are coexpressing *Tlr9* and *Clec7a* (top area) and clusters which express either one or neither (bottom area).

Among the innate compartment, 12 subsets were identified and annotated on the basis of canonical markers (fig. S3B), including monocyte-derived macrophages denoted as monocyte-macrophage (Mo-Mac: *Fcgr1*, *Adgre1*, *H2-Ab1*, and *Ly6c2*), alveolar macrophages (*Fcgr1*, *Adgre1*, *H2-Ab1*, *Itgax*, and *Siglecf*), DCs (*Zbtb46* and *H2-Ab1*), plasmacytoid DCs (pDCs: *H2-Ab1*, *Siglech*, and *Ly6a*), neutrophils (*S100a8*, *S100a9*, and *Ly6g*), monocytes (*Ly6c2* and *Ccr2*), natural killer (NK) cells (*Klrb1c*, *Prf1*, and *Ncr1*), and ILC2/3 (*Thy1*, *Gata3*, *Rora*, and *Rorc*). Comparing the subsets across treatment and time, K3-SPG induced robust changes to cell proportions at day 3 postadministration. From day 1 to day 3, the induction of Mo-Mac and monocytes, accompanied by the reduction of NK cells and alveolar macrophages, was identified. The identified macrophage clusters excluding alveolar macrophages were largely absent in PBS-treated mice (Fig. 2A). Among the identified clusters, Mo-Mac appeared to be most induced after K3-SPG treatment. Therefore, we conducted gene ontology (GO) analysis of Mo-Mac populations using the top 100 up-regulated genes in day 3 pretreated K3-SPG lungs compared to its PBS counterpart (Fig. 2B). Both KEGG pathways and GO biological processes results suggested enriched cytokine-related pathways and chemotaxis, which may reflect the induction of Mo-Mac in the lungs of K3-SPG–treated mice. Further, clusters were categorized on the basis of *Tlr9* and *Clec7a* (Dectin-1) expression (fig. S3, C and D) due to the role of Dectin-1 for uptake and TLR9 for innate immune activation by K3-SPG (*27*). Coexpression of both genes were associated with the up-regulation of monocyte-recruiting gene *Ccl2*, as well as chemokines *Cxcl9* and *Cxcl10*, and IFN-stimulated genes (ISGs), most prominently in the Mo-Mac cluster. This finding aligns with previously conducted GO analysis, prompting us to further investigate the Mo-Mac cluster in more detail. Even in the absence of *Tlr9* and *Clec7a* coexpression, we observed up-regulation of ISGs in neutrophils and *Ifng* in NK cells, suggesting that the priming of TLR9 and Dectin-1 coexpressing cells may be followed by a secondary immune response through cytokine/chemokine that could be crucial for the mechanisms of K3-SPG–mediated protection (Fig. 2C).

We further investigated the adaptive immune cells and identified no clear changes to cell proportions, aside from the slight induction of *Cd4*^+^ *Foxp3*^+^ regulatory T cells, as well as *Tnf*- and *Ifng*-expressing γδ T cells (fig. S3A). In contrast, when analyzing nonimmune cells, K3-SPG administration led to the expansion of endothelial cells, along with simultaneous reduction of club cells and fibroblasts (fig. S3E). These demonstrate that K3-SPG not only affects myeloid compartment but potentially also activates the adaptive and nonimmune cell compartments that altogether contribute to enhancing host resistance in acute lethal infection.

### Generation of transient CD11c^+^CD11b^+^ macrophage population with sustained activation states

To further investigate the Mo-Mac population in the lungs, we used the flow cytometry approach. On the basis of the lung immune cell gating strategy that was previously reported (*33*), the CD45^+^MHC-II^+^CD64^+^ cells were distinguished as macrophage. There are two different tissue resident macrophages in the lung, and we denoted CD11c^+^CD11b^−^ cells as alveolar macrophages (AMΦ) and CD11b^+^CD11c^−^ as interstitial macrophages (IMΦ).

In a time-course experiment from day 1 to day 30 posttreatment, lung macrophage populations were significantly changed in K3-SPG–treated mice (Fig. 3A). AMΦ displayed significant reduction in numbers after K3-SPG treatment, most prominent at day 3 (Fig. 3B). This was accompanied with a simultaneous induction of IMΦ and an intermediate population, which we denoted as double-positive (DP) MΦ due to the coexpression of CD11b^+^CD11c^+^. While IMΦ and DP MΦ did not persist in the lungs for sustained period, macrophage activation markers such as CD80, CD86, and major histocompatibility complex II (MHC-II) showed sustained up-regulation up to 21 days (Fig. 3C). To elucidate the function of K3-SPG–induced macrophages for immunoprophylactic efficacy, we performed adoptive transfer of macrophages isolated from day 3 pretreated donors into naïve recipients. When macrophages were transferred directly into the lung parenchyma via intratracheal administration, recipients of IMΦ and DP MΦ from K3-SPG–treated donors demonstrated enhanced protection, which was significant compared to PBS-treated control mice, even with lethal infection (2LD_50_ of PR8) (Fig. 3D). These findings confirm that K3-SPG–induced macrophages are crucial players of early innate immune response against lethal influenza infection.

**Fig. 3.**
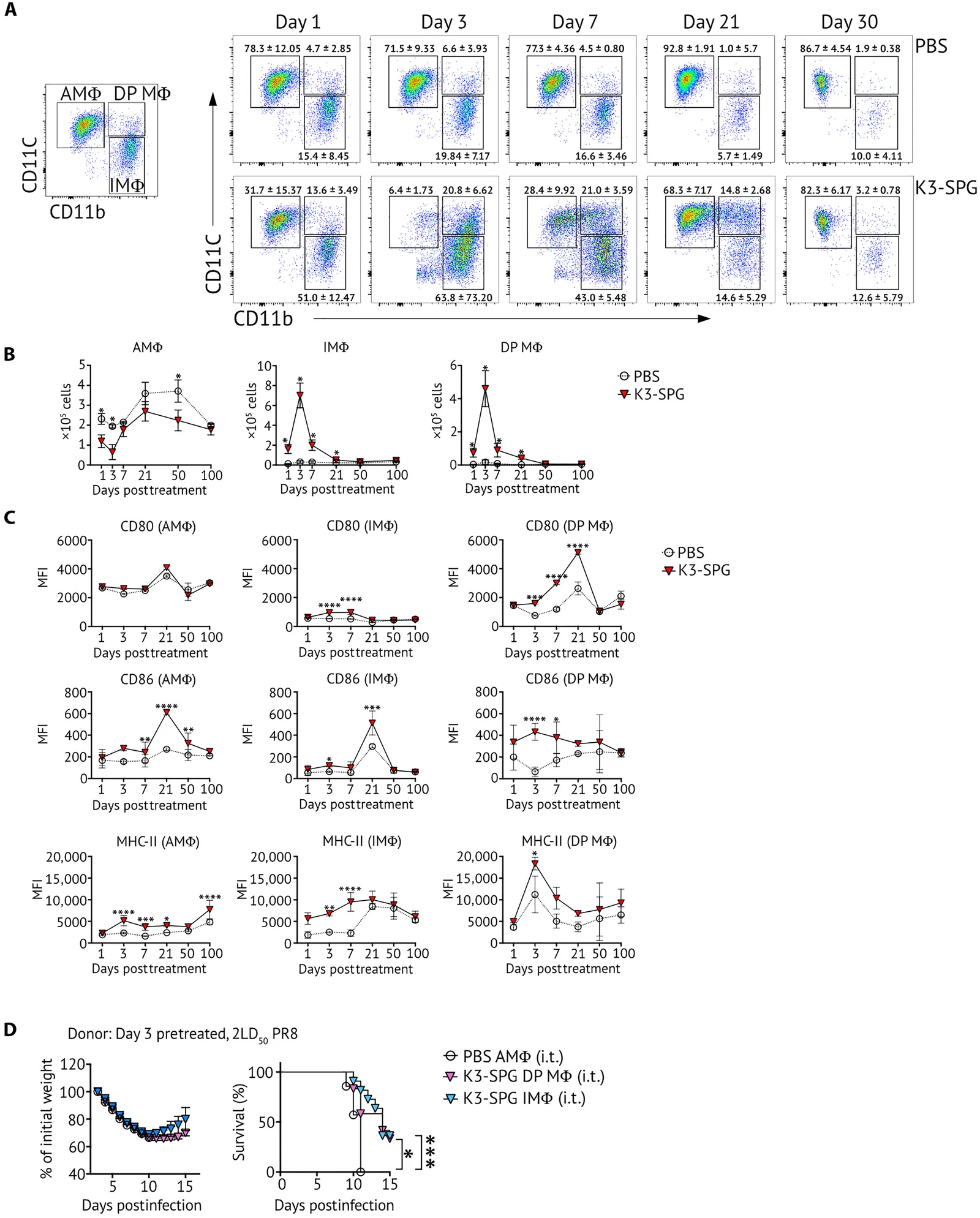
Generation of transient CD11c^+^ CD11b^+^ macrophage population with sustained activation states. (**A**) Lung macrophage population after gating on I-A/I-E^+^ CD64^+^. Flow cytometry plots are representative of at least two independent experiments for each time point. (**B**) The change of macrophage numbers in the lungs from day 1 to day 100 posttreatment. (**C**) Macrophage activation by measurement of median fluorescence intensity (MFI) of CD80 (top), CD86 (middle), and MHC-II (bottom), separated by macrophage subsets from day 1 to day 100 posttreatment. Data were pooled from at least two independent experiments, with *n* ≥ 3 for each independent experiment. (**D**) Adoptive transfer of 1 × 10^5^ cells per recipient from day 3 pretreated donors. Individual macrophage subsets were isolated from lung cells and transferred intratracheally (1 × 10^5^ cells per recipient). At 24 hours posttransfer, mice were infected with PR8 (2LD_50_) and monitored up to 15 dpi. Data are pooled from two independent experiments, with the total number of *n* = 10 to 12 for K3-SPG recipients and *n* = 6 for PBS recipients. Mann-Whitney tests, *P ≤ 0.05 (B); multiple comparisons with two-way analysis of variance (ANOVA), *P ≤ 0.05, **P < 0.01, ***P < 0.001, and ****P < 0.0001 (C); survival comparison conducted with Kaplan-Maier log-rank (Mantel-Cox) test, *P ≤ 0.05 and ***P < 0.001 (D).

A previous report by Chakraborty *et al.* (*34*) demonstrated that lipopolysaccharide (LPS)–challenged mice induced a DP MΦ-like population that showed fluctuating Siglec-F expression, suggesting that AMΦ is a possible source for these cells. While Siglec-F is not restricted to AMΦ, they remain an important marker for murine AMΦ (*35*). Therefore, we monitored the Siglec-F expression in the three macrophage subsets (Fig. 4A). From day 1 to day 7, AMΦ remained largely Siglec-F^+^, IMΦ remained Siglec-F^−^, and DP MΦ demonstrated fluctuating Siglec-F expression, where it down-regulated Siglec-F at day 3. To investigate the identity of DP MΦ, we used an in vivo cell membrane–labeling approach as previously reported (*36*). Mice were administered PKH26 intranasally, and after a resting period of 5 days, intranasal administration of PBS or K3-SPG was performed. Lung tissue was analyzed at 3 days post-PBS or post–K3-SPG administration. First, we confirmed that PKH26 labeling was not spilled into the bloodstream or the spleen (fig. S4A). While PKH26 successfully stained ∼80% of cells in the BALF, only 10% of cells in the lung tissue were stained, which composed primarily of macrophages. These data suggest that PKH26 is a suitable approach for staining and tracking of resident cells in the lung airspace. Our data suggest that DP MΦ is composed of both resident AMΦ and monocyte-derived IMΦ (Fig. 4B) that is consistent with fluctuating Siglec-F expression.

**Fig. 4.**
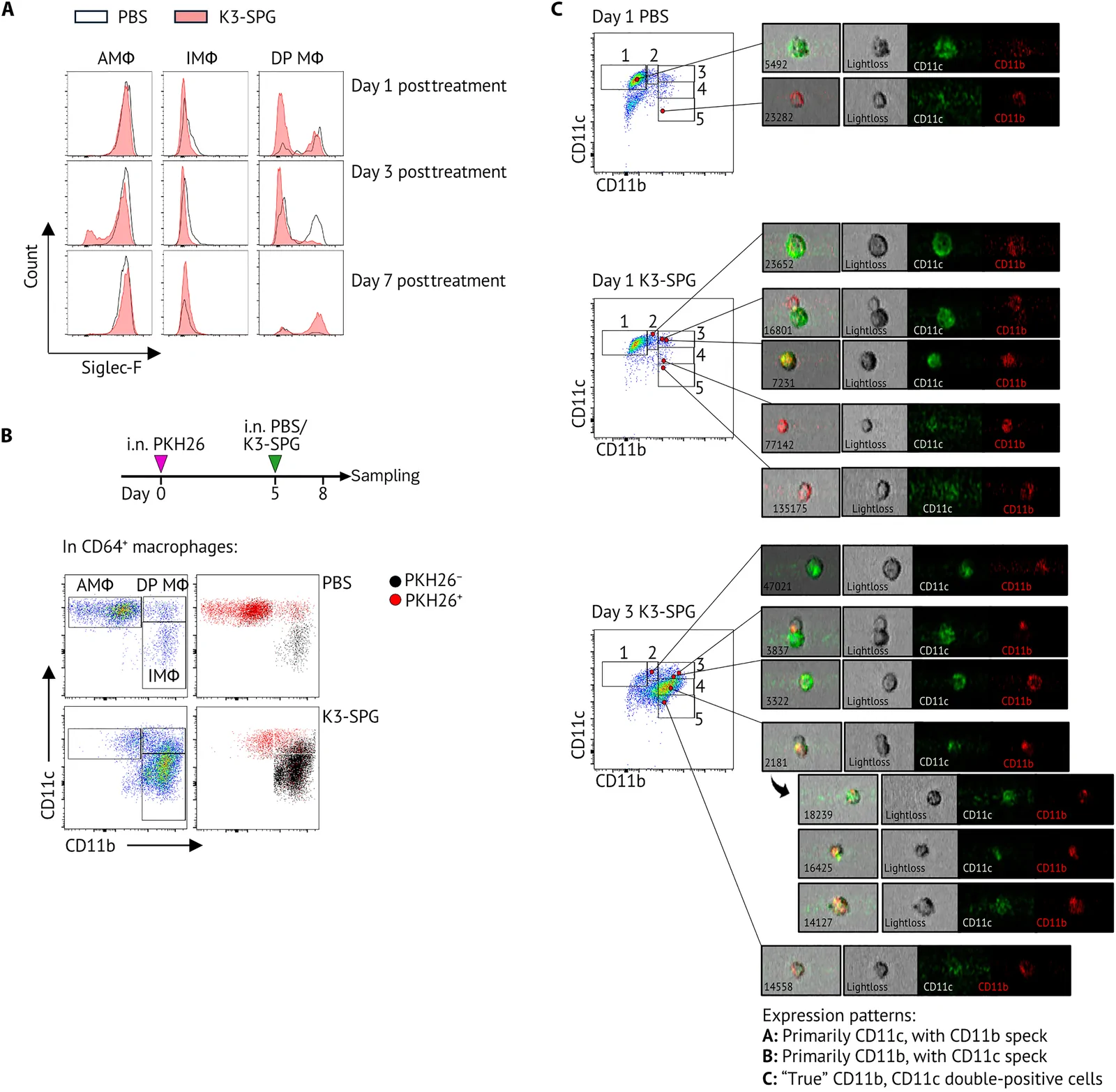
DP MΦ demonstrates unique CD11b and CD11c expression on the surface by real-time cytometer imaging. (**A**) Siglec-F expression in AMΦ, IMΦ, and DP MΦ from day 1 to day 7 posttreatment. Flow cytometry plots shown are representative of at least three independent experiments, with *n* ≥ 3 for each independent experiment. (**B**) In vivo PKH26 labeling to track induction of DP MΦ. Top displays the experimental timeline of PKH26 labeling. Bottom presents the flow cytometry plots depicting the movement of PKH26 signals in PBS- and K3-SPG–treated mice. Data shown are representative of two independent experiments, *n* ≥ 3 for each independent experiment. (**C**) Imaging of surface expression of CD11b and CD11c on lung macrophages. From flow cytometry plot, gates were categorized on the basis of the expression pattern of CD11c and CD11b as follows: (1) CD11c^hi^CD11b^low^ (AMΦ1), (2) CD11c^hi^CD11b^int^ (AMΦ2), (3) CD11c^hi^ CD11b^hi^ (DP MΦ), (4) CD11b^hi^ CD11c^low^ (IMΦ1), and (5) CD11b^hi^ CD11c^int^ (IMΦ2). Day 1 PBS images depict baseline signals from imaging channels for AMΦ (gate 1) and IMΦ2 (gate 5). In day 3 K3-SPG, extension from gate 4 displays the expression pattern of imaged cells denoted as A, B, and C. Imaging was performed on the BD DiscoverS8 Cell Sorter platform. Images shown are representative of two independent experiments, *n* = 2 for each independent experiment.

Recent studies explore the mechanism of cell-cell interactions and communications which may involve transfer of cellular components from a donor cell to a recipient cell, such as trogocytosis and extracellular vesicle–mediated processes (*37*–*39*). We investigated the involvement of these processes to further understand how DP MΦ were generated. Using the BD DiscoverS8 platform for simultaneous single-cell analysis and imaging, we investigated the expression pattern of CD11b and CD11c on lung macrophages obtained at 1 and 3 days posttreatment (Fig. 4C). Because of background signals, cells with negative marker expression does not display clear cellular morphology, while a positive marker expression is characterized by clear cellular morphology in the imaging channel. When we further focused on MHC-II^+^CD64^+^ macrophage population by CD11c and CD11b expression, we could identify AMΦ as (gate 1) CD11c^hi^CD11b^low^ (AMΦ1) and (gate 2) CD11c^hi^CD11b^int^ (AMΦ2), DP MΦ as (gate 3) CD11c^hi^ CD11b^hi^, and IMΦ as (gate 4) CD11b^hi^ CD11c^int^ (IMΦ1) and (gate 5) CD11b^hi^ CD11c^low^ (IMΦ2). We could observe the sequential generation of DP MΦ (gate 3) which appear to be heterogeneous populations composing of both singlets and interacting doublets despite the inclusion of FSC/SSC singlet gate (Fig. 4C). At day 3, three patterns of CD11b/CD11c expression were observed, which are primarily CD11c with CD11b specks on the surface (pattern A), primarily CD11b with CD11c specks (pattern B), and “true” CD11b, CD11c DP cells (pattern C). These findings suggest that DP MΦ is generated from both the resident AMΦ and induced IMΦ. The exact mechanism of gaining CD11b or CD11c from one cell to another is not yet elucidated, although our findings suggest that transfer of cellular component may be a key process required for the development of transient macrophage population, which may be crucial for viral protection.

To determine the drivers for induction of IMΦ and DP MΦ, we explored the role of cytokine signaling pathways. Because of enriched chemotactic pathways and up-regulation of monocyte-recruiting gene *Ccl2* in our single-cell and GO analyses datasets, we measured CCL2, also known as monocyte chemoattractant protein-1 (MCP-1), production in BALF alongside inflammatory cytokine TNF-α, known to regulate monocyte function (fig. S4B) (*40*). Induction of both molecules suggested a monocyte recruitment mechanism for generation of macrophages in the lungs. In *Tnf*α^−/−^ mice, IMΦ and DP MΦ were not induced after K3-SPG treatment (fig. S4C). A previous study demonstrated that CpG ODN administration induces IMΦ and triggers differentiation of recruited inflammatory monocytes into IMΦ in the lung (*41*). To confirm the contribution of inflammatory monocytes for induction of IMΦ and DP MΦ, we evaluated the recruitment of inflammatory monocytes (Ly6C^hi^CD11b^+^) from the spleen to the lungs after K3-SPG administration using *Tnf*α^−/−^ mice. In wild-type mice, a significant increase of inflammatory monocytes in the lungs at 3 days postadministration was observed, but this phenomenon was abrogated in *Tnf*α^−/−^ mice (fig. S4D), demonstrating the potential role of TNF-α in the induction of monocyte-derived macrophages in the lungs. In addition, when we investigated the splenic inflammatory monocyte population, both groups of mice showed slight reduction. Reports show that CCL2 (MCP-1) is highly involved in monocyte recruitment from the spleen to sites of inflammation (*41*, *42*). While it is often associated with TNF-α, induction of CCL2 in the BALF from *Tnf*α*^−/−^* mice was not significantly different from *Tnf*α ^+/−^ mice (fig. S4E). These data suggest that other signaling molecules may work together with TNF-α. Furthermore, when *Tnf*α^−/−^ mice were challenged with 2LD_50_ PR8 virus at 3 days following K3-SPG treatment, we identified TNF-α dependence (fig. S4F). These results suggest that TNF-α–mediated macrophage inductions are key for K3-SPG–induced immunoprophylactic efficacy.

### Thy1^+^ non–T cell ILCs undergo epigenetic reprogramming and contribute to long-term protection

In our scRNA-seq data, we also observed the increased number of ILCs and NK cell–related subsets. Therefore, we focused on non–T cells *Thy1*^+^ subsets, comprising conventional NK cells, NKT cells, and a small population of ILCs in our scRNA-seq dataset (Fig. 5A). The distinct gene expression of each cluster is presented in Fig. 5B. While there was no clear separation of NK and ILC cluster in the UMAP, we observed preferential expression of genes such as *Il2ra* (CD25), *Kit* (c-Kit and CD117), and *Gzmc* (granzyme C) in the ILC cluster, confirming their distinct identities which probed us to investigate further. To measure the induction of specific ILC subsets, time-course flow cytometry was performed using the gating strategy as shown in Fig. 6A, which includes the use of lineage staining [CD5, CD3, FcεRIα, B220, Gr-1, T cell receptor β (TCRβ), TCRγδ, and TER-119] and transcription factors—T-bet for ILC1, GATA3 for ILC2, and RORγt for ILC3. Although numerous studies include CD11b, CD11c, and NK1.1 in ILC-negative lineage cocktail, we used a modified panel to avoid loss of ILC1s and activated ILC subsets, which can gain expression of noncanonical genes thus serving as potential activation markers (fig. S5A). We confirmed the distinct identities of conventional NK cells (NK1.1^+^ CD11b^+^ without lineage exclusion) and ILC1 by surface marker up-regulation of Sca-1, Thy1, and T-bet following K3-SPG administration (fig. S5B). Significant induction of ILC1 (T-bet^+^ NK1.1^+^) and ILC3 (RORγt^+^ Sca-1^+^) was observed in a time-dependent manner, where both subsets peaked at day 3 posttreatment (Fig. 6B). In contrast, ILC2 showed a slight increase at 6 hours and day 7 postadministration; however, no clear time-dependent activation was observed. Consequently, we measured the induction of ILC1 and ILC3-related effector cytokines IFN-γ and IL-17A (Fig. 6C). We identified that TNF-α is crucial for macrophage and ILC induction (Fig. 6D), suggesting that TNF-α is an indispensable mediator for initiating the protective immune responses by K3-SPG.

**Fig. 5.**
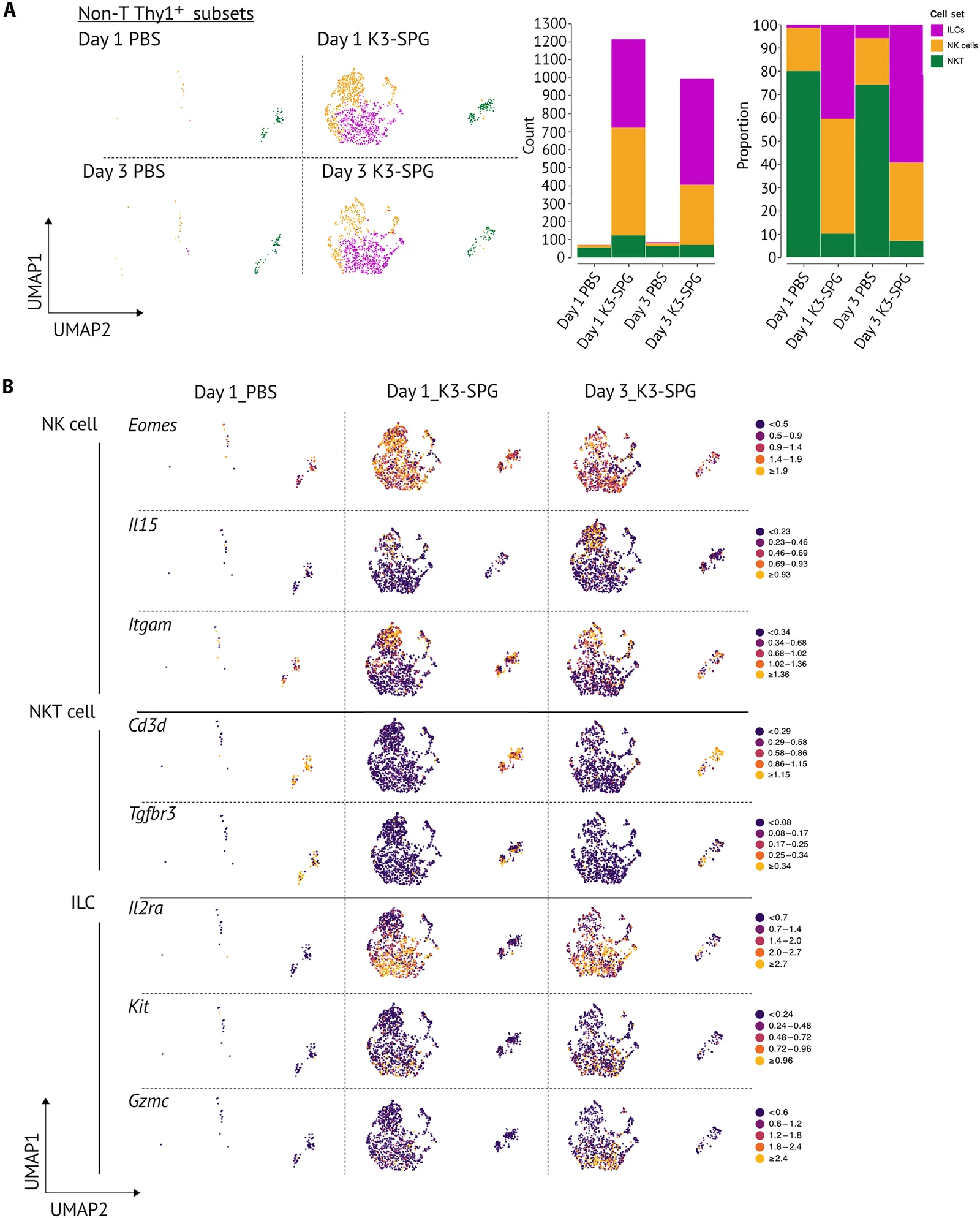
Robust induction of Thy1^+^ non–T cell lung ILCs has distinct gene expression from conventional NK cells. (**A**) Left: scRNA-seq UMAP displaying only *Thy1*^+^ NK cell–related subset by exclusion of cells expressing *Cd19* (B cells), *Col1a1* (fibroblasts), *Fcgr1* (macrophages), *Trgv* and *Trgd* (γδ T cells), and *Cd3e* (bulk T cells). Resulting UMAP consists of only three clusters. Right: Stacked bar graph represents the count data of each identified cluster among the *Thy1*^+^ NK cell–related subsets. (**B**) Categorical embedding of some cluster-specific genes for NK cell, NKT cell, and ILCs.

**Fig. 6.**
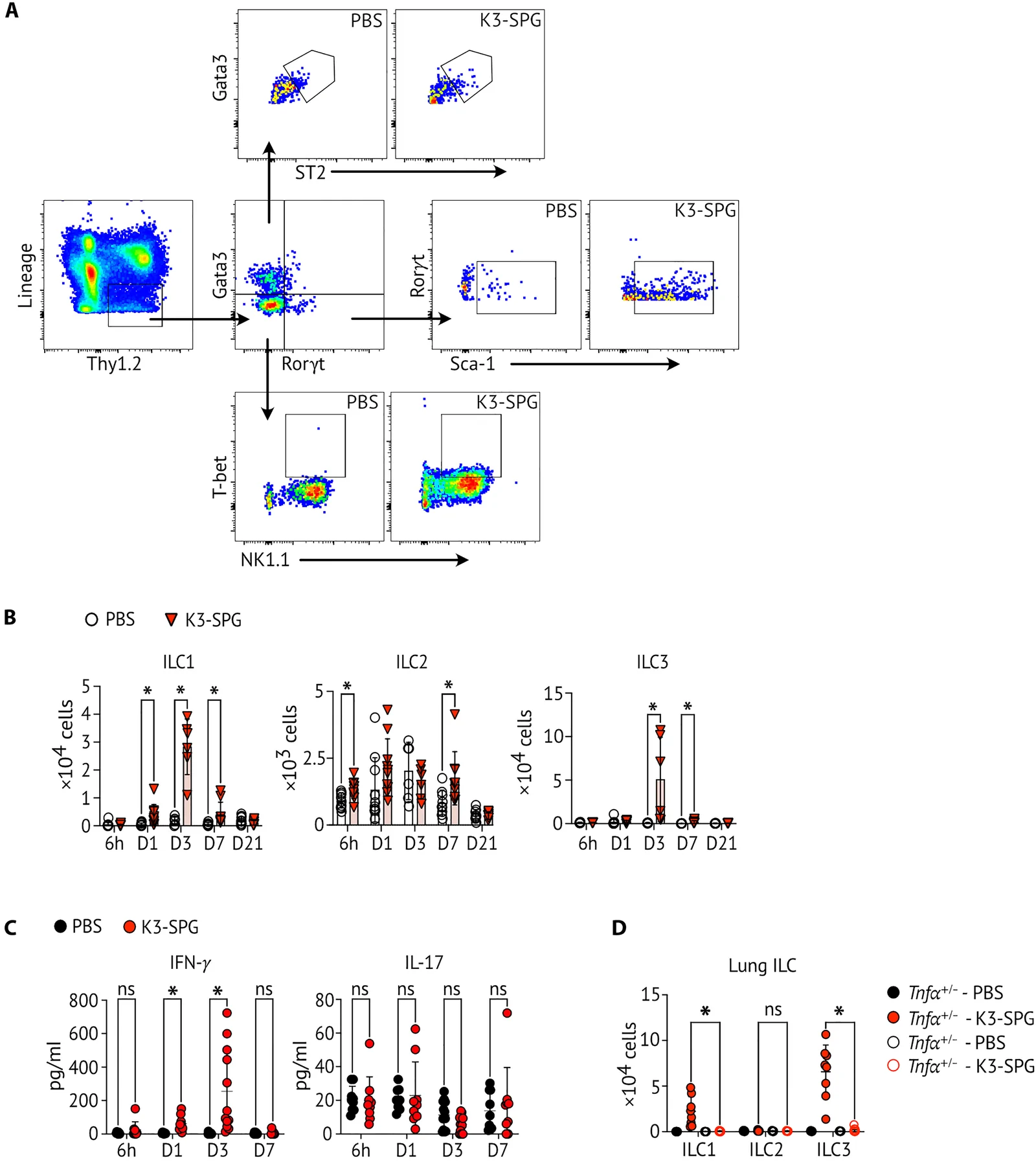
ILC1 and ILC3 are induced by K3-SPG via TNF-α. (**A**) Flow cytometry gating strategy for studying ILC subsets in the lungs by transcription factor staining. (**B**) Number of ILC1, ILC2, and ILC3 after PBS or K3-SPG treatment, from 6 hours to day 21 posttreatment. Data pooled from two independent experiments, with *n* ≥ 3 for each independent experiment. (**C**) Measurement of IFN-γ and IL-17A in BALF from day 1, day 2, and day 3 pretreated mice. Measurement was conducted by a multiplex assay system. (**D**) The number of ILC subsets in PBS and K3-SPG day 3 pretreated *Tnf*α^+/−^ and *Tnf*α^/−^ mice. Data pooled from two independent experiments, with *n* = 3 to 4 for each independent experiment. Multiple comparisons Mann-Whitney test, *P ≤ 0.05 [(B) to (D)].

ILC subsets returned to untreated levels 21 days after K3-SPG treatment. Therefore, we investigated the epigenetic modifications underlying long-lasting immunity. To investigate whether ILCs are epigenetically modified in the long term, we investigated chromatin accessibility in day 21 pretreated ILCs. To overcome the inability of intranuclear staining for cell sorting, we used a simplified gating strategy using only surface markers (fig. S6A). Unexpectedly, ILC1 remains largely unchanged between PBS and K3-SPG groups as demonstrated in the principal components analysis (PCA) plot (Fig. 7A) and Homer motif enrichment analysis, where no known motifs and only de novo motifs with high false positives could be identified, likely due to having only 25 differentially accessible regions (DARs). Among 21 DARs, innate immune-related genes such as *Stat3*, *Ccl3*, *Slc11a1*, and *Tnfaip3* were identified as more open chromatin regions in K3-SPG_ILC1. On the other hand, ILC3 and ILC2 display a similar profile, with ILC3 from K3-SPG–treated mice suggesting some level of differentiation compared to the PBS counterpart. By visualizing chromatin region peaks in IGV (Integrative Genomics Viewer), we confirmed chromatin regions in transcription factors which identify ILC identity (fig. S6B).

**Fig. 7.**
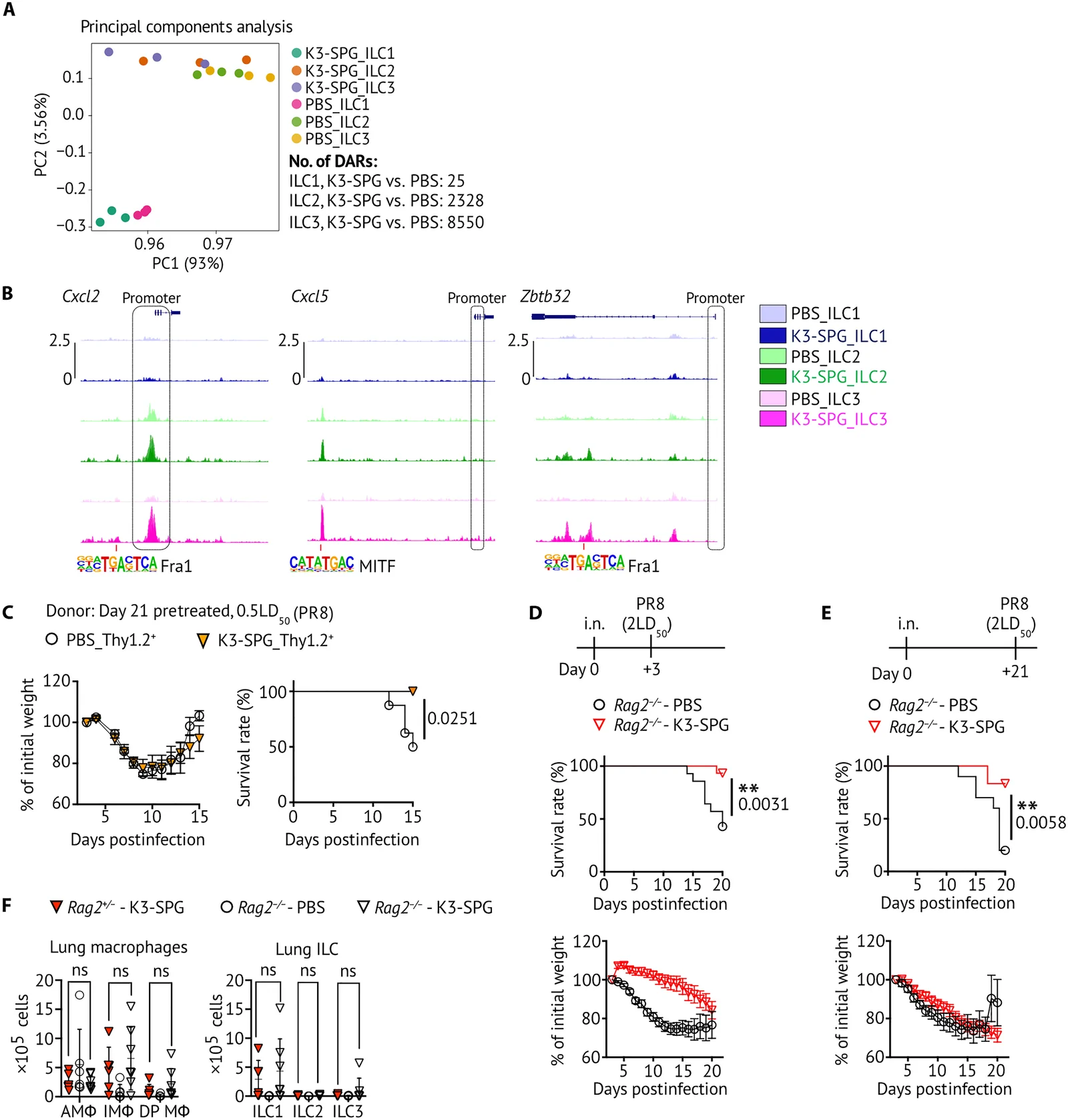
ILCs sustain epigenetic modifications and protected naïve mice up to 21 days. (**A**) PCA plot between day 21 pretreated ILCs from PBS- or K3-SPG–treated mice (one experiment with *n* = 3). (**B**) IGV peaks demonstrating open chromatin regions in K3-SPG–treated ILC2 and ILC3, in chemotaxis-related genes (*Cxcl2* and *Cxcl5*) and memory marker *Zbtb32*. (**C**) Adoptive transfer of Thy1.2^+^ cells from day 21 pretreated PBS or K3-SPG mice. A total of 1 × 10^5^ cells were transferred via intravenous injection in 200 μl of PBS, to naïve recipients. Sublethal infection was performed 24 hours after cell transfer, and mice were monitored daily for survival and body weight loss up to 15 dpi. Data are pooled from two independent experiments, with the total number of recipient mice *n* = 8. (**D**) Sublethal PR8 challenge to *Rag2*^−/−^ mice treated 3 days before infection. Mice were monitored for survival and body weight up to 20 dpi. Data are pooled from two independent experiments, with *n* = 6 to 8 for each independent experiment. (**E**) Sublethal PR8 challenge to *Rag2*^−/−^ mice treated 21 days before infection. Mice were monitored for survival and body weight up to 20 dpi. Data are pooled from two independent experiments, with *n* = 5 to 6 for each independent experiment. (**F**) Number of macrophages and ILCs in *Rag2*^+/−^ and *Rag2*^−/−^ mice at 3 days posttreatment. Data are pooled from two independent experiments, with *n* = 3 to 4 for each independent experiment. Mann-Whitney test (F); survival comparison conducted with Kaplan-Maier log-rank (Mantel-Cox) test, **P < 0.01 [(C) to (E)].

In de novo motif analysis, 9 and 14 motifs were identified among ILC2 (table S1) and ILC3 (table S2), respectively. The three motifs which demonstrated highest correlation to peak data using IGV is as depicted in fig. S6C. Open chromatin regions in genes typically related to ILC1 and ILC3 functions, such as *Ifng*, *Tnf*, and *Il17a*, could not be observed. In contrast, Fra1 and MITF motif sequences were found at open chromatin regions in upstream of the promoters for chemokine ligands *Cxcl2* and *Cxcl5* (Fig. 7B). Both ligands bind to CXCR2 receptor, which modulates immune cell recruitment to inflamed lungs (*43*). We also identified epigenetically modified *Zbtb32* gene, more often associated with NK and B cell memory (*44*, *45*), correlated to the Fra1 motif. Collectively, our findings reveal that CpG ODN-primed ILC activation may sustain regulatory or effector roles against viruses via noncanonical epigenetic reprogramming.

Upon determining that ILCs are epigenetically altered at 21 days posttreatment, we sought to investigate whether they are effector cells for long-term protection. As a strategy to overcome the limitations of cell numbers, we performed adoptive transfer of Thy1.2^+^ cells from wild-type day 21 pretreated donors and observed full protection (Fig. 7C). To eliminate the possibility of T cell contribution for K3-SPG–mediated protection, *Rag2*^−/−^ mice were pretreated 3 and 21 days before infection. K3-SPG pretreated *Rag2*^−/−^ mice demonstrated >80% survival (Fig. 7, D and E), while the absence of *Rag2* gene did not affect induction of both macrophages and ILCs (Fig. 7F).

### K3-SPG protects mice in a TLR9-dependent manner

CpG ODN, via TLR9 signaling, is known to drive production of inflammatory cytokines, such as TNF-α (*46*), which we have identified controls the initiation of protective immune responses. This prompted us to investigate whether K3-SPG truly acts in a TLR9-dependent manner for macrophages and ILC induction and viral protection. We pretreated *Tlr9*^−/−^ and *Tlr9*^+/−^ mice with PBS or K3-SPG at 3 days before flow cytometry analyses. Both macrophage and ILC induction demonstrated TLR9 dependence (Fig. 8A), in addition to activation of macrophages by surface expression of CD80, CD86, and MHC-II (Fig. 8B). When we challenged day 3 K3-SPG pretreated *Tlr9*^−/−^ mice (Fig. 8C), we observed complete abrogation of K3-SPG–mediated protection. Further, we investigated the role of Dectin-1 in the uptake of K3-SPG (Fig. 8D). In the absence of Dectin-1, induction of IMΦ and ILC3 showed slight reduction without statistical significance. Simultaneously, ILC1 induction was completely abrogated. These findings demonstrate the complexity of K3-SPG–induced innate activation via TLR9 that is sufficiently potent to protect against lethal flu infection.

**Fig. 8.**
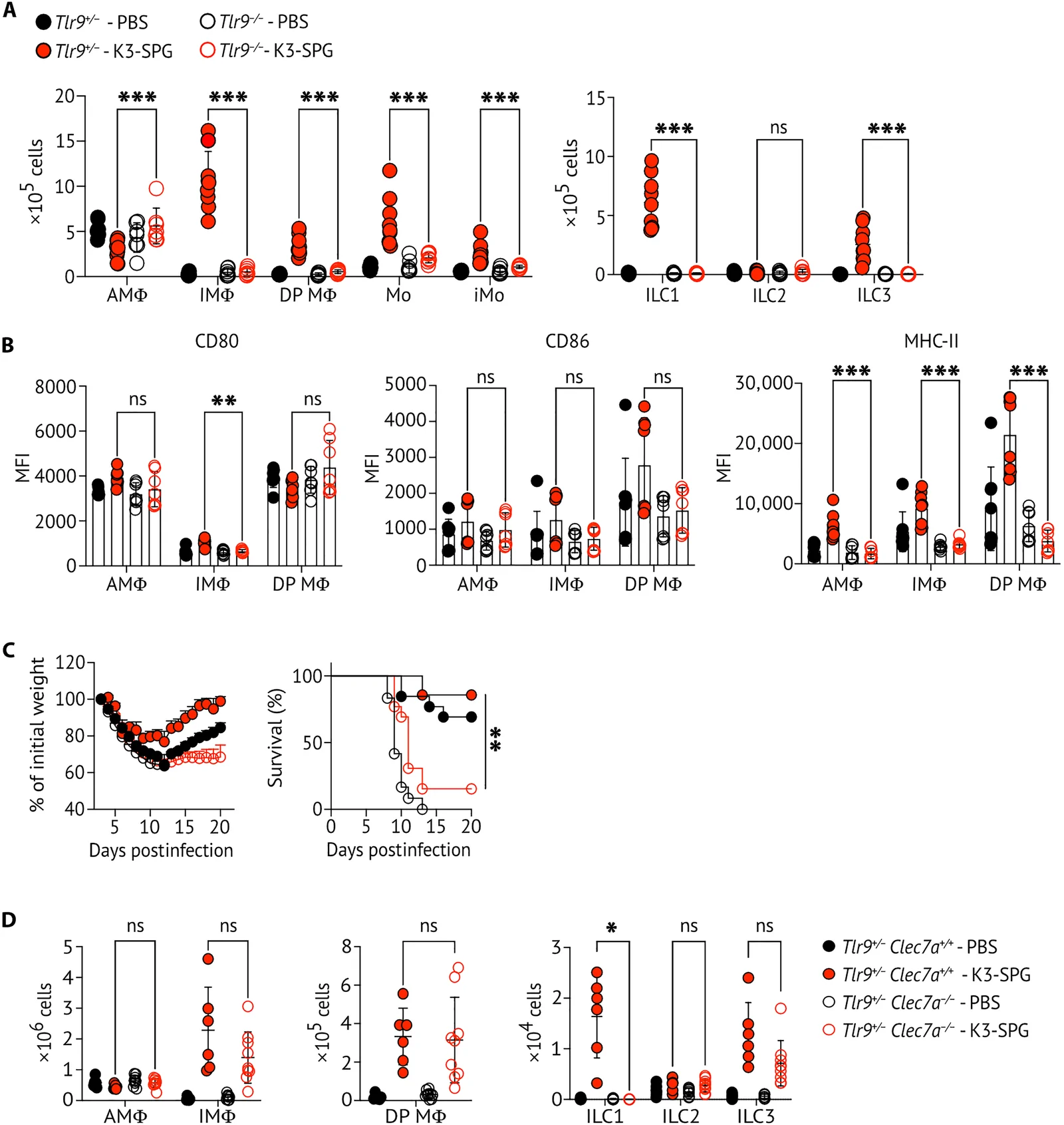
K3-SPG–mediated cellular induction and in vivo protection are dependent on TLR9-signaling. (**A**) Number of macrophages, monocytes (Mo), inflammatory monocyte (iMo), and ILCs after K3-SPG treatment to *Tlr9*^+/−^ and *Tlr9*^−/−^ mice, obtained at day 3 posttreatment. (**B**) Macrophage activation (MFI) by CD80, CD86, and MHC-II after K3-SPG treatment to *Tlr9*^+/−^ and *Tlr9*^−/−^ mice, measured at day 3 posttreatment. Data are pooled from two independent experiments, with *n* = 3 to 4 for each independent experiment. (**C**) Lethal PR8 challenge to *Tlr9*^+/−^ and *Tlr9*^−/−^ mice treated 3 days before infection. Mice were monitored daily for survival and body weight loss up to 20 dpi. Data are pooled from two independent experiments, with *n* = 6 to 7 for each independent experiment. (**D**) Number of macrophages and ILCs after K3-SPG treatment to *Tlr9*^+/−^ *Clec7a*^+/+^ and *Tlr9*^+/−^ *Clec7a*^−/−^ mice, obtained at day 3 posttreatment. Data are pooled from two independent experiments, with *n* = 3 to 4 for each independent experiment. Multiple Mann-Whitney test, *P ≤ 0.05, **P < 0.01, and ***P < 0.001 [(A), (B), and (D)]; survival comparison conducted with Kaplan-Maier log-rank (Mantel-Cox) test, **P < 0.01 (C).

The expression of TLR9 in mouse and humans is distinct. Therefore, we sought to assess whether K3-SPG could potentially induce innate memory in human cells. Using an in vitro training assay (fig. S7), we stimulated human leukocytes with K3-SPG or zymosan depleted (positive control) for 24 hours. Upon primary stimulation, adjuvants were washed out, and cells were rested in media for 24 or 72 hours, followed by LPS challenge (10 ng/ml) for 24 hours. Our data suggest that K3-SPG has the potential to activate and induce memory in human circulating leukocytes. While the magnitude of innate induction appears lower than zymosan depleted, these data confirm its potential for clinical application.

## DISCUSSION

This study demonstrates that K3-SPG is capable to confer potent immunotherapeutic effects in mice, enhancing host resistance against lethal respiratory virus infection for prolonged duration. Aligning with our aim to address the gap between pathogen emergence and vaccine availability in the next pandemic, our findings show the promising potential of K3-SPG for clinical applications in the future. Our study reports the complexation of CpG ODN into the β-glucan molecule itself, and not by simple coadministration or generation of liposome formulations, as previously reported (*47*). First, we identified that K3-SPG–induced immunoprophylaxis, while long-lasting, offers waning protection with time. This waning phenomenon could be correlated to the gradual reduction of macrophage and ILC numbers in the lungs, despite the sustained enhanced expression of activation markers in the former. The extend of epigenetic reprogramming in these cells would be a crucial factor to consider identifying the cause of waning protection. In addition, in contrast to numerous papers, we showed that intranasally administered β-glucan alone was unable to confer protection against lethal flu infection. This contrasting results from available reports may stem from the differences in dose, route of administration, and type of β-glucan used (*23**,* *24**,* *48*). This raises the possibility that upon changing the source of β-glucan in the composition of K3-SPG, we may further enhance the efficacy, including the duration of protection.

Mechanistically, we proposed that primary response by macrophages leads to a secondary response involving the induction of rare lung ILC1 and ILC3. In the early phase, the transient CD11b^+^CD11c^+^ DP MΦ subset exhibited fluctuating Siglec-F expression, where its expression was down-regulated at 3 days after K3-SPG administration. This reduction of Siglec-F expression has been reported in prior reports of innate immune memory and is proposed to be one of the phenotypes associated with trained AMΦ (*34*). The trained macrophage can be characterized with enhanced phagocytic and efferocytic functions and correlated to epigenetic reprogramming, such as enriched transcription factor KLF4 which has been associated to regulate lung macrophage function (*34*). Several studies have proposed that AMΦ can also be replenished by bone marrow–derived macrophages, and these macrophages are reported to express both CD11b and CD11c (*49*, *50*). On the other hand, while there are studies demonstrating lung ILC induction in the context of infection (*51*, *52*), reports on adjuvant-induced ILCs remain scarce. Research on ILCs have expanded in recent years, uncovering their effector functions conferring antiviral and antitumor effects (*53*–*55*). The ongoing controversy behind ILCs stems from their shared surface and gene expression with the NK cell counterpart, making them challenging to approach. As reported in prior studies, ILCs can be rapidly induced in the early phases of infection (*51*, *56*), retain epigenetic modifications, and acquire memory-like responses for as long as 4 months postinfection (*57*, *58*). This aligns with our findings where although we observed induction of ILCs as early as 1 day posttreatment, its protective functions were still evident at 21 days posttreatment.

Our data demonstrate that, rather than inducing conventional antiviral mechanisms that directly suppresses viral replication, intranasal administration of K3-SPG promotes a disease tolerance phenotype that generates long-term protection against respiratory viruses. This is supported by improved lung pathology without reduction of viral titers. Furthermore, although TNF-α is often regarded as proinflammatory whose activity must be tightly regulated, our findings uncover a previously unrecognized regulatory function of the cytokine, establishing the role of TLR9-induced TNF-α that is indispensable for the establishment of the observed tolerogenic phenotype. A similar phenomenon was also reported in a prior study by Khan *et al.* (*24*), where the authors demonstrated that β-glucan alone is capable to reprogram host innate immunity, characterized by bone marrow–derived neutrophils which enhanced disease tolerance, leading to preservation of lung integrity without affecting viral burden. Together, these studies shed light on the approach to use innate stimulatory adjuvants to improve host resistance by limiting immunopathology, which presents an attractive strategy when faced with emerging or unknown pathogens.

Beyond mechanistic insights, our proposition to use K3-SPG or other adjuvant compounds as a strategy to confer temporary protection until a vaccine is available requires much consideration, especially for safety and route of administration as we translate our study from mouse to human application. A study in 1999 (*59*) demonstrated that repeated systemic administration of CpG ODN with a 2-week interval for up to 2 months showed no noticeable adverse effects, measured by hepatomegaly, splenomegaly, and blood smear analyses. Furthermore, both components used in complexation of K3-SPG has been clinically evaluated for its safety in humans, in phase 1 clinical trials for advanced lung cancer (K3) (*60*), and in clinical application of SPG as a cancer immunotherapy adjuvant (*61*). As we consider clinical application, addressing the difference between intranasal route of administration is crucial. Our data suggest that entry of K3-SPG into the lung tissue is necessary to induce protective effects. However, in humans, intranasal sprays often limit the distribution of the drug to the nasal mucosa. To address this gap, the establishment of aerosol systems for adjuvant administration may be important. In addition, now available resources enable us to explore the potential efficacy only by stimulation of peripheral blood mononuclear cells or whole blood. It would be beneficial to perform in vitro training models using cells isolated from human BALF. Moreover, the expression of TLR9 in humans and mouse is distinct. In the former, TLR9-expressing cells are primarily B cells and pDCs; however the expression is much more broadly distributed in mouse. Using a protocol optimized for β-glucan training (*62*), our data demonstrated potential of K3-SPG to confer memory phenotype in human circulatory leukocytes. To assess the true efficacy of K3-SPG, it would be ideal to optimize the in vitro training assay for intracellular adjuvant such as K3-SPG. Bridging this gap between murine and human studies would greatly contribute to progressing the research field.

Some key questions still remain to be unanswered which warrants further investigations. First, the duration and magnitude of adjuvant-mediated protection, although statistically significant compared to PBS-treated mice, offers moderate protection at 50 days postadministration and low protection at 100 days postadministration. Therefore, the number of DP MΦ and IMΦ populations may contribute to the duration of protection. However, other cell types, including ILCs and other immune or nonimmune cell populations, could also contribute to the maintenance of long-term immunoprophylaxis. In clinical application, a sustained high protection is ideal, and it is a challenge to achieve in the absence of antigen. Further experiments assessing changes to the dose, regimen, and combination of administration routes could be explored in future studies. In addition, although we observed that adoptive transfer of Thy1.2^+^ cells and K3-SPG–treated *Rag2*^−/−^ mice conferred protection against influenza virus challenge, the direct contribution of ILCs to K3-SPG–mediated immunoprophylactic effects remains unclear. Future studies using ILC-deficient mice or ILC depletion approaches is required to address this gap. These approaches would also enable us to address the potential trans-differentiation phenomenon from lung prevalent ILC2 into effector ILC1 and ILC3 subsets (*52*, *63*–*65*).

Together, we identified a CpG ODN–β-glucan complex adjuvant as a potential emergency immunotherapy to be applied in the next pandemic. Acting in an antigen-independent manner, we observed the protective ability of K3-SPG against lethal influenza infection. This study highlights a unique, noncanonical type of innate memory in the lungs, promoting a tolerogenic phenotype involving two key innate immune cell subsets. While the proposition to use K3-SPG globally to achieve the 100 Days Mission is a feat to achieve, this study provides the fundamental evidence of its efficacy and will lead the direction for future studies in preparation for clinical application.

## MATERIALS AND METHODS

### Study design

Sample sizes for each independent experiment were determined on the basis of the minimum number required for statistical analysis. No predefined rules for stopping data collection were applied, and all animal experiments were conducted in accordance with institutional guidelines. No explicit criteria for exclusion of data or outliers were applied, and all obtained data points are included in the figures. Primary endpoints for infection studies are survival and body weight loss until recovery of surviving mice (20 dpi). Primary endpoints for noninfection study include immune cell profiling by flow cytometry, transcriptomic analysis by scRNA-seq and assay for transposase-accessible chromatin (ATAC) sequencing, and evaluation of cytokine responses. Except Fig. 1C (subcutaneous) and sequencing experiments, all experiments were independently repeated at least twice.

### Mice

In all experiments, female 6-week-old wild-type C57BL/6J mice were used, unless stated otherwise. C57BL/6J mice were purchased from NIHON CLEA. *Tnf*α (*66*), *Rag2* (*67*), and *Tlr9* (*23*) knockout mice were maintained in our animal facility. Breeding, colony maintenance, and genotyping were performed in-house according to established protocols. All animal experiments were conducted following the institutional guidelines of the Institute of Medical Science, The University of Tokyo (approval no. A2025IMS140-01). No mice were excluded on the basis of predefined criteria. Randomization was performed only before treatment.

### Preparation and stimulation of human leukocytes

Leukocytes were obtained from five healthy adult volunteers with informed consent. All experiments using human leukocytes were approved by the Institutional Review Board of the Institute of Medical Science, The University of Tokyo. Leukocytes were prepared by red blood cell (RBC) lysis of whole blood, performed by 1:10 whole blood:lysis buffer ratio. After incubation for 15 min, cells were centrifuged and washed in complete RPMI [RPMI 1640 supplemented with 10% (vol/vol) fetal bovine serum (FBS), penicillin/streptomycin, 0.01 mM Hepes, MEA-NEAA, 1 mM sodium pyruvate, and 0.055 mM 2-mercaptoethanol]. Cells were seeded at a density of 1 × 10^5^ cells per well and stimulated with K3-SPG (10 μg/ml) or zymosan depleted (Invivogen, 5 μg/ml). Supernatants were subjected to enzyme-linked immunosorbent assay (ELISA) for TNF-α (R&D Systems, #DY210) and IL-6 (R&D Systems, #DY206). Experiment was conducted with approval and according to the institutional guidelines of the Institute of Medical Science, The University of Tokyo (approval no. 2024-15-0620).

### Mouse treatment and infection

For all experiments, adjuvant was diluted in PBS, and PBS was used as the control treatment. For adjuvants, 10 μg each of K3-SPG, K3-dA_40_ (Aji Bio-Pharma Oligos), SPG (Invivogen), curdlan (Invivogen), zymosan depleted (Invivogen), zymosan (Invivogen), poly(I:C) (HMW) (Invivogen), and ODN1668 (Invivogen) was used in this study, where applicable. The synthesis of K3-SPG was as previously described (*27*). The volume of each dose differs on the basis of the administration route as follows: 30 μl (intranasal), 50 μl (subcutaneous), and 200 μl (intravenous via tail vein using a 29G 12.7-mm U-100 Insulin syringe from BD Biosciences). All intranasal administration were performed under anesthesia.

### Influenza virus challenge

For influenza A infection experiments, mice were intranasally challenged with clinically isolated A/Puerto Rico/8/1934 (H1N1) strain (*27*) at 2LD_50_ (1 × 10^2^ PFU) for lethal experiments and 0.5LD_50_ (25 PFU) for sublethal experiments, unless stated otherwise.

### SARS-CoV-2 challenge

Breeding and maintenance of hACE2/TG mice were conducted according to previously published procedures (*68*). For infection experiments, mice were anesthetized [100 μl per animal; medetomidine (20 μg/ml), midazolam (600 μg/ml), and butorphanol (1 mg/ml)] and intubated using an otoscope and intubation platform. The ancestral SARS-CoV-2 strain (SARS-CoV-2/UT-NCGM02/Human/2020/Tokyo; NCGM02) was administered intratracheally at 2 × 10^3^ median tissue culture infectious dose. Body weight and survival were recorded daily for up to 14 dpi. Animals exhibiting apparent wasting were euthanized following body weight measurement and were classified as nonsurvivors. All SARS-CoV-2 infection studies were performed in enhanced BSL-3 containment at the Tsukuba Primate Research Center of the NIBN, in accordance with institutional standard operating procedures (the animal protocol number is DSR02-44).

### Collection and processing of tissues

The preparation of lung and spleen single-cell suspensions was according to Miltenyi-Biotec lung and spleen dissociation kits, respectively. Briefly, collected tissues were added into MACS C-tubes containing a mixture of buffer S, enzyme D, and enzyme A, according to the manufacturer’s protocol. After 30 and 15 min of mechanical dissociation (GentleMACS Dissociator; Miltenyi Biotec), respectively, cell suspensions were filtered through a 70-μm cell strainer and centrifuged for 10 min at 300*g*, 4°C. The supernatant was discarded, and RBC lysis was performed using 1× RBC lysis buffer (eBioscience; Invitrogen). After RBC lysis, cells were washed, filtered once more, and counted using an automatic cell counter (Facscope B, Curiosis) for subsequent analysis by flow cytometry and/or further processing for RNA sequencing.

### BALF collection

BALF was collected by washing the lungs with 0.5 ml of sterile cold PBS, twice, for a total volume of 1 ml. Briefly, an incision was made at the upper region of the trachea, and the insertion of a plastic cannula (Terumo, Tokyo, Japan) was performed. BALF obtained was centrifuged at 400*g* for 4 min (4°C), and the supernatant was collected for cytokine measurement by ELISA.

### Plaque assay for viral titer measurement of BALF

Virus titers were measured by plaque assay using MDCK (Madin-Darby canine kidney) cells. MDCK cells were provided by the Japanese Collection of Research Bioresources cell bank.

Cells were seeded in six-well plates and grown to 90 to 100% confluence. BALF samples were diluted 10-, 100-, and 10,000-fold in sterile PBS. Cell monolayers were washed once with PBS and inoculated with 200 μl of diluted BALF. Plates were incubated for 1 hour at 37°C with gentle tilting every 15 min to allow virus adsorption and even distribution. Then, cells were removed, and wells were overlaid with 2 ml of overlay medium consisting of 0.6% agarose (SeaKem ME agarose, Lonza, #50011) in 2× MEM (Sigma-Aldrich, #M0275) supplemented with bovine serum albumin (Sigma-Aldrich, #A7409), 0.1% NAHCO_3_ (Gibco, #25080-094), and acetylated trypsin (10 μg/ml; Sigma-Aldrich, #T6763). Plates were incubated at 37°C in 5% CO_2_ for 40 to 48 hours in an inverted position. Virus inactivation was performed by ultraviolet exposure for at least 45 min. Staining was performed in 0.1% crystal violet (Wako, #038-17792), and plaques were visualized with a Keyence fluorescence microscope (BZ-X1000). Plaques were manually counted, and graphs were generated on GraphPad Prism v.11.0.1.

### Micro-CT imaging

At 3 days before virus infection, mice were intranasally treated with PBS or K3-SPG, followed by 2LD_50_ influenza virus challenge. Before adjuvant treatment (naïve) and at 4, 6, and 9 dpi, the lungs of infected mice were imaged by using an in vivo three-dimensional micro-CT scanner (CosmoScan GXII; Rigaku CT Lab GX). Briefly, under isoflurane anesthesia, the mice were placed in the image chamber and scanned at high speed for 8 s at 90 kV, 88 μA, and field of view of 36 mm. After scanning, the lung images were reconstructed by using the CosmoScan Database software of the micro-CT (Rigaku Corporation) and analyzed using the manufacturer-supplied software. Experiment was conducted once with *n* = 5 per treatment group. Figure is represented with representative image.

### Flow cytometry

Immune profiling of lung and spleen immune cell subsets was performed with BD LSR II flow cytometer (BD Biosciences, San Jose, CA, USA). For each sample, at least 1 × 10^6^ cells were used for staining. Visualization of live cells was achieved with the LIVE/DEAD Fixable Aqua Dead Cell Stain Kit (Invitrogen, San Diego, CA, USA). After dead cell staining, Fc blocking was performed using TruStain FcX (BioLegend, clone: 93) followed by staining with surface marker antibodies for respective tissues as follows:

To study lung immune cell subsets, the cells were stained with anti–I-A/I-E (BioLegend, clone: M5/114.15.2), anti–Ly-6C (BioLegend, clone: HK1.4), anti-CD90.2 (Thy1.2; BioLegend, clone: 30-H12), anti-CD11c (BioLegend, clone: N418), anti-CD11b (BioLegend, clone: M1/70), anti-CD45 (BioLegend, clone: 30-F11), anti-CD80 (BioLegend, clone: 16-10A1), anti-CD86 (BioLegend, clone: GL-1), anti-CD64 (Fc𝛾RI; BioLegend, clone: X54-5/7.1), anti-CD24 (BioLegend, clone: M1/69), anti-CD170 (Siglec-F; BioLegend, clone: S17007L), anti-Ly-6G (BioLegend, clone: 1A8), and anti-Ly6A/E (Sca-1; BD Biosciences, clone: D7). To study lung ILCs, cells were surface stained with anti-CD45 (BioLegend, clone: 30-F11), anti-CD5 (BioLegend, clone: 53-7.3), anti-CD3e (BioLegend, clone: 145-2C11), anti-CD45R (B220; BioLegend, clone: RA3-6B2), anti-TER-119 (BioLegend, clone: TER-119), anti-TCRβ (BioLegend, clone: H5-597), anti-TCRγδ (BioLegend, clone: GL3), anti-FcεRIα (BioLegend, clone: MAR-1), anti–Gr-1 (BioLegend, clone: RB6-8C5), anti-Thy1.2 (BioLegend, clone: 30-H12), anti-ST2 (BioLegend, clone: DIH9), anti–Sca-1 (BioLegend, clone: D7), anti-NK1.1 (BioLegend, clone: PK136), and anti-CD127 (BioLegend, clone: A7R34). After surface staining, Foxp3 transcription factor staining kit (eBioscience, #00-5523-00) was used according to the manufacturer’s guidelines. Cells were stained with transcription factors as follows: anti-Gata3 (BioLegend, clone: 16E10A23), anti–T-bet (BioLegend, clone: 4B10), and anti-Rorγt (BioLegend, clone: AFKJS-9). To study immune cell subsets of the spleen, the cells were stained with anti–Ly-6C (BioLegend, clone: HK1.4), anti-NK1.1 (BioLegend, clone: PK136), anti–I-A/I-E (BioLegend, clone: M5/114.15.2), anti–Ly-6G (BioLegend, clone: 1A8), anti-TCRβ (BioLegend, clone: H57-597), anti-CD19 (BioLegend, clone: 6D5), anti-CD8a (BioLegend, clone: 53-6.7), anti-CD45 (BioLegend, clone: 30-F11), anti-CD11b (BioLegend, clone: M1/70), anti-CD11c (BioLegend, clone: N418), anti-CD90.2 (Thy1.2; BioLegend, clone: 53-2.1), anti-CD4 (BioLegend, clone: RM4-5), and anti-F4/80 (BioLegend, clone: BM8).

For sorting, BD Influx, BD FACSAria, BD FACSMelody, or BD FACSDiscover S8 (BD Biosciences, San Jose, CA, USA) and CytoFLEX SRT (Beckman Coulter) were used for sorting of macrophages and ILCs. All flow cytometry data were analyzed with FlowJo software (Tree Star).

### Adoptive transfer experiment

After sorting of target population in Dulbecco’s modified Eagle’s medium supplemented with 10% FBS, cells were washed with cold PBS and counted using a hemocytometer. Adjustment of cells was performed on the basis of transfer route—(i) intratracheal: 1 × 10^5^ cells in 50 μl and (ii) intravenous: 1 × 10^5^ cells in 200 μl. Medium used for suspending cells and for cell transfer was sterile PBS. Intratracheal administration was performed as previously described (*69*). Briefly, mice were anesthetized and placed upright on a board by their incisors. A 1-ml syringe and a flexible pipette tip were inserted into the trachea, and 50 μl of cell suspension was injected directly into the lungs. For intravenous administration, awake mice were placed under a heat lamp for vein dilation before injection. After ∼5 min, mice were set on a restrainer. Using a 0.5-ml 29G syringe, 200 μl of cell suspension was injected directly into the vein.

### ELISA for cytokine and chemokine responses

Measurement of cytokines and chemokines from BALF samples was conducted by using DuoSet ELISA kits (R&D Systems), according to the manufacturer’s instructions. TNF-α (#DY410), IL-6 (#DY406), IFN-γ (#DY485), IL-17 (#DY421), and CCL2 (MCP-1) (#DY479) were measured.

### Assay for transposase-accessible chromatin with sequencing

ATAC sequencing of sorted ILC populations collected 21 days after K3-SPG or PBS treatment. Briefly, lungs from PBS- or K3-SPG–treated mice were collected and processed using a Miltenyi lung dissociation kit as reported in previous sections. ILCs were sorted with an average sort of efficiency of ≥68% using CytoFLEX SRT (Beckman Coulter). Cells were kept in CELLBANKER1 in −80°C for 1 week before thawing, cell counting, and sequencing.

After freeze and thaw, 5 × 10^3^ to 4.8 × 10^4^ cells were obtained, and downstream processing was performed as previously described (*70*). Cells were lysed in cold lysis buffer [10 mM tris-HCl (pH 7.4), 10 mM NaCl, 3 mM MgCl_2_, and 0.1% IGEPAL CA-630] on ice for 10 min. After centrifugation, nuclei pellets were resuspended with 50 μl of transposase reaction mix [25 μl of Tagment DNA buffer (Illumina), 2.5 μl of Tagment DNA enzyme (Illumina), and 22.5 μl of water], incubated at 37°C for 35 min, and were purified with a MinElute PCR Purification Kit (QIAGEN). After the optimization of polymerase chain reaction (PCR) cycle number using SYBR Green I nucleic acid gel stain (Takara Bio), transposed fragments were amplified using NEBNext High Fidelity 2× PCR Master mix and index primers and were purified with a MinElute PCR Purification Kit (QIAGEN). Library DNA was size-selected (240 to 360 bp) using BluePippin (Sage Science, Beverly, MA, USA). Sequencing was performed using a HiSeq1500 or HiSeq2500 (Illumina) with a single-read sequencing length of 50 bp. Sequences were aligned to mouse genome sequences (mm10) using Bowtie2 (version 2.5.2) (*71*) with the default settings. Peaks were called using MACS3 (version 3.0.1) (*72*) with a *q* value cutoff of <0.01 and model settings. The catalog of all peaks called in any sample was produced by merging all called peaks that overlapped by at least 1 bp using the bedtools (version 2.31.1) merge function. We then removed the regions that were detected in only one sample. A total of 53,350 peaks were detected and used as map files for downstream processing. The bedtools map function was used to count the reads in each region of the catalog using the bed files of each sample. A read count matrix was used for the normalization and detection of differentially accessible peaks using DESeq2 (version 1.42.1) (*73*). PCA was performed with the R package prcomp using a normalized count. For motif analysis, findMotifsGenome.pl. of Homer (version 4.11.1) (*74*) was used with the -size 200 -mask option, and all catalog regions were used as the background. For the annotation of peaks, annotatePeaks.pl of Homer was used with default settings.

Visualization of peaks in the genome was performed using IGV (UC San Diego) (*75*).

### Single-cell RNA sequencing

scRNA-seq of whole lungs collected 1 day and 3 days after K3-SPG or PBS treatment (*n* = 3) was performed using the 10X Genomics Chromium System. Lungs were processed using a Miltenyi lung dissociation kit, as reported in previous sections. Cell suspensions were subjected to dead cell removal by using a Dead Cell Removal kit (Miltenyi Biotec) according to the manufacturer’s protocol. A total of 1.6 × 10^4^ cells were used for downstream processing and sequencing.

Libraries were generated using the Chromium GEM-X Single Cell 3′ Reagent Kits v4 (10X Genomics). Single-cell sequencing of the libraries was performed by KOTAI Biotechnologies Inc. (Osaka, Japan) with an MGI DNBSeq-G400 sequencer, followed by downstream quality control and filtering of the dataset. Cell Ranger files were generated using cellranger-8.0.1 by matching to the reference genome GRCm39-2024-A, provided by KOTAI Biotechnologies Inc. Recovery of cells per sample ranged from approximately 1 × 10^4^ to 2 × 10^4^ cells. Mean reads per cell were reported to be 36,681 to 88,865, indicating moderate to high coverage across samples. Furthermore, median genes detected per cell ranged from 3449 to 3916 with Q30 scores > 90% across samples.

### scRNA-seq analysis

The scRNA-seq dataset was explored and visualized using the Trailmaker platform by Parse Biosciences (*76*). Filtered matrices from Cell Ranger were uploaded onto Trailmaker. Further filtering of the samples was performed on the basis of Trailmaker’s recommended parameters based on their automated pipeline. Briefly, mitochondrial content threshold was set using an absolute threshold method, with maximum allowed fractions ranging from 2.5 to 3.9%, depending on the sample. Linear regression of number of genes and UMIs was used to detect outliers, with significance thresholds of approximately 4 × 10^−5^ to 9 × 10^−5^. Potential doublets were excluded by applying sample-specific threshold ranging from 0.23 to 0.43. Data integration and log normalization were performed using Seurat with Harmony. A total of 23 principal components were identified. Clustering was performed via the Leiden method.

First, whole lung UMAP was generated and broadly annotated on the basis of key canonical genes, such as *Cd3e* for T cells and *Cd19* for B cells. *Ptprc*-low or the absence of *Ptprc* gene was used for identification of nonimmune cells. Upon identifying these three clusters, remaining cells (myeloid and other innate cells) were subsetted and reclustered in the same manner as described above. In the innate cell UMAP, 12 clusters were identified as shown in Fig. 2A. Separately, nonimmune cells were subsetted and reclustered for investigating the change in proportion of nonimmune subsets, as depicted in fig. S2E. T cells and B cells were also analyzed in the same manner as depicted in fig. S2A. Here, we show each individual subsetted data and not the whole lung UMAP.

### GO analyses

All gene expression data were obtained from the respective scRNA-seq datasets. GO analyses were conducted on Metascape (*77*).

### Statistics

Statistical analyses were performed using GraphPad Prism version 10.5.0 and version 11.0.1 (GraphPad Software, San Diego, CA). Data are presented as mean ± SD for cell populations and median fluorescence intensity (MFI) figures and mean ± SEM for time-course body weight loss data. The specific statistical test and statistical significance cutoff point (*P* value) for the corresponding experiment are listed in the figure legend of corresponding figures. Briefly, Mann-Whitney was used when comparing between two groups (*P ≤ 0.05). When there is one independent variable, a multiple comparisons nonparametric test was used to compare three or more groups between two treatment groups (*P ≤ 0.05). When there are two independent variables (e.g., time-course analysis), two-way analysis of variance (ANOVA) was used. Correction for the multiple comparisons test was conducted with the Holm-Šidák method. Survival comparison was conducted using the Kaplan-Maier log-rank (Mantel-Cox) test. For statistical analysis of human samples of five donors, a paired *t* test was used to compare between negative and experimental groups.

### Human participants or animal subjects (IRB/IACUC approvals)

#### Human participants

All experiments using human leukocytes were approved by the Institutional Review Board of the Institute of Medical Science, The University of Tokyo (approval no. 2024-15-0620).

#### Animal subjects

All animal experiments classified as specific pathogen–free and BSL-2 for influenza challenge studies were conducted following the institutional guidelines of the Institute of Medical Science, The University of Tokyo (approval no. A2025IMS140-01). All SARS-CoV-2 infection studies were performed in enhanced BSL-3 containment at the Tsukuba Primate Research Center of the NIBN, in accordance with institutional standard operating procedures (approval no. DSR02-44).
