# Supplementary material for: Long-term immunoprophylaxis by TLR9 ligand mediates macrophage-ILC cross-talk against respiratory viral infection
Source: Sci Adv. 2026 Oct 9;12(41):eaeh6480. doi: 10.1126/sciadv.aeh6480 (PMC13651865; doi:10.1126/sciadv.aeh6480)
Supplement: Supplementary file 1 [file sciadv.aeh6480_sm.pdf]

Science Advances
AAAS

Supplementary Materials for

Long-term immunoprophylaxis by TLR9 ligand mediates macrophage-ILC cross-talk against respiratory viral infection

Asuka Joy Tobuse *et al.*

Corresponding author: Kouji Kobiyama, kkobiyama@health.ucsd.edu; Ken J. Ishii, kenishii@ims.u-tokyo.ac.jp

Sci. Adv. **12**, eaeh6480 (2026)
DOI: 10.1126/sciadv.aeh6480

This PDF file includes:

Figs. S1 to S7
Tables S1 and S2

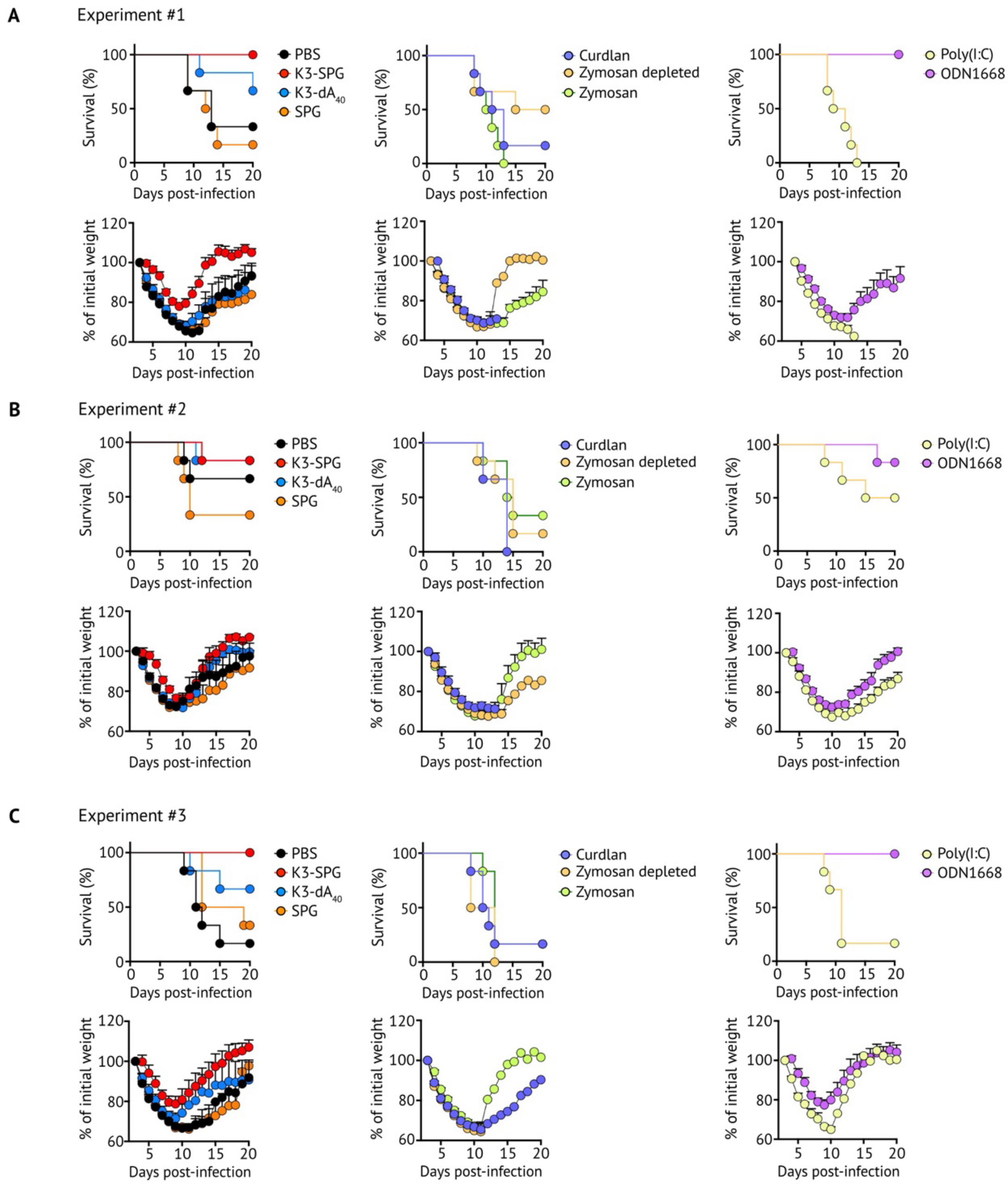

Fig. S1. K3-SPG and TLR9 agonist confers superior protection compared to β-glucan and TLR3 agonist.

Individual experiment data from Fig. 1A. Each independent experiment (n=6-7) is labeled from A through C.

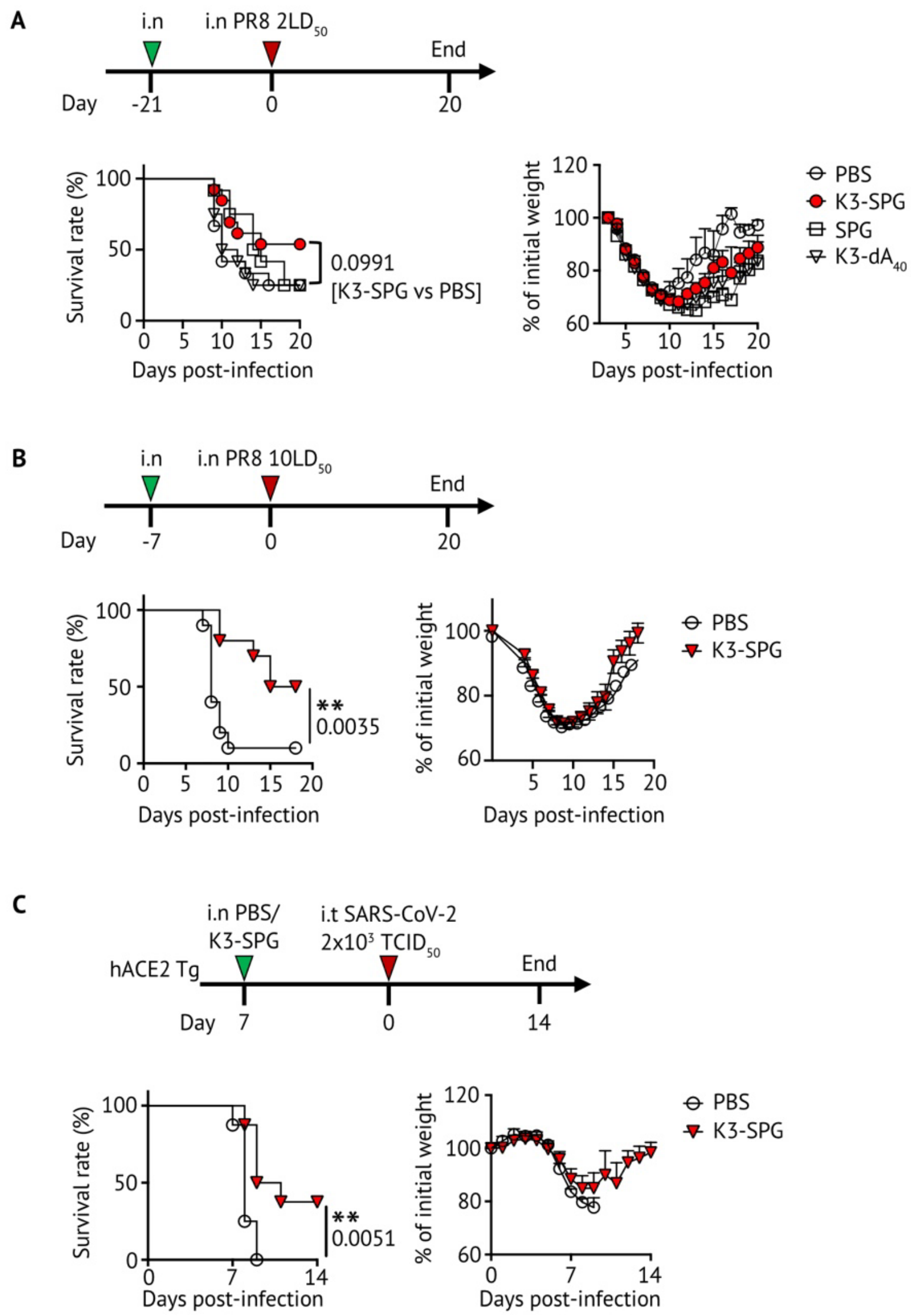

Fig. S2. Long-term protection is mediated by K3-SPG.

(A) C57BL/6J mice (female, 6-wk) were intranasally treated with PBS, K3-SPG, K3-dA$_{40}$, and SPG. At 21 days after treatment, lethal PR8 infection was conducted. Mice were monitored daily for survival and body weight loss up to 20 dpi. Data is pooled from two independent experiments, n=12. (B) PR8 challenge at 5x higher lethal dose in day 7 pre-treated PBS or K3-SPG mice

(n=10). Mice were monitored daily for survival and body weight loss up to 20 dpi. (C) SARS-CoV-2 challenge using hACE2 Tg mice (n=8). Intranasal treatment of PBS or K3-SPG was performed 7 days prior to virus challenge of $2x10^3$ TCID$_{50}$. Mice were monitored daily for survival and body weight loss for 2 weeks. Survival comparison conducted with Kaplan-Maier log-rank (Mantel-Cox) test.

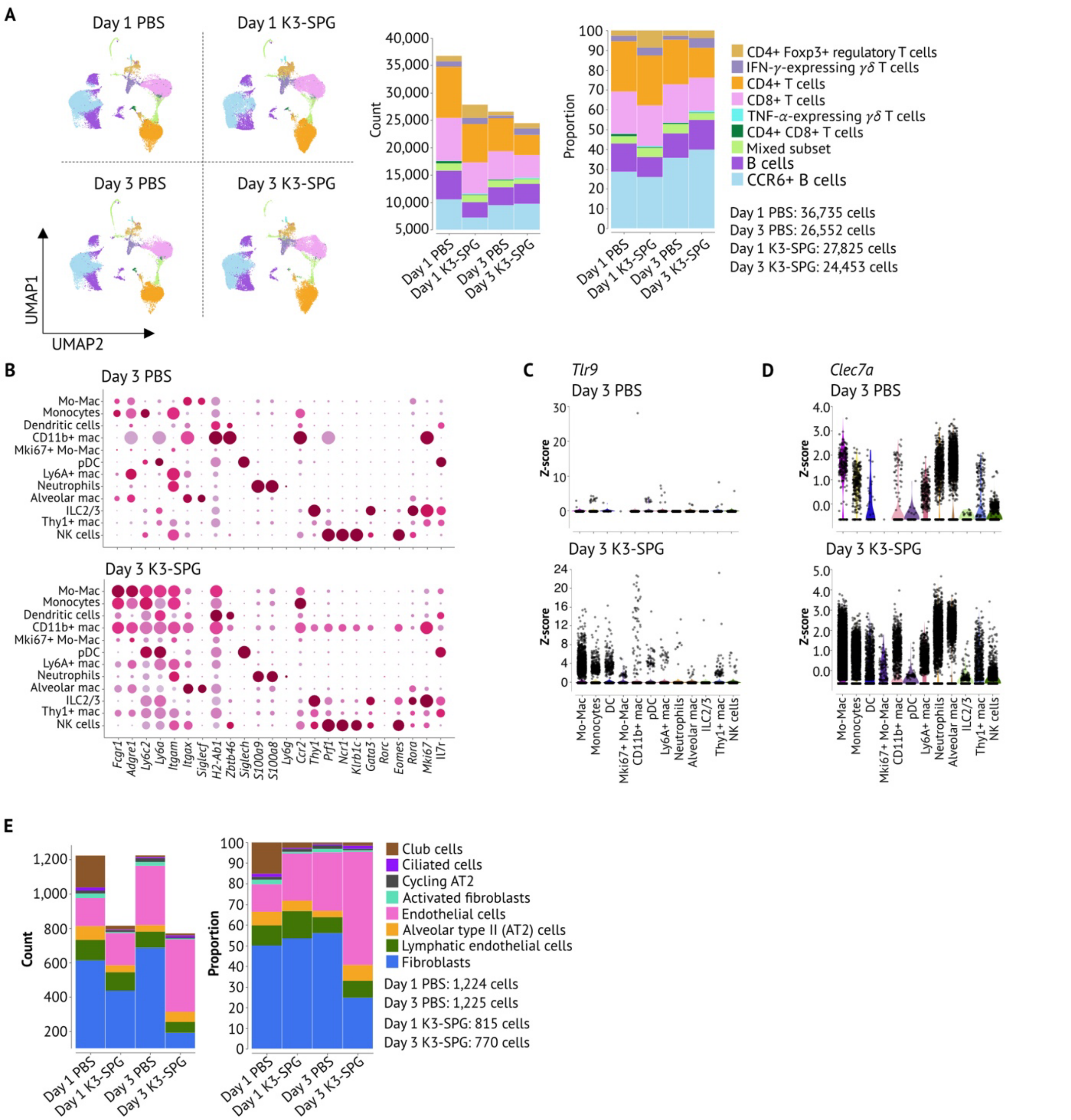

Fig. S3. Single-cell RNA sequencing reveals complex changes to lung cell diversity.
(A) Left panel shows the UMAP presenting adaptive immune cell compartment of the lungs generated by *Cd19* and *Cd3e* expression. A total of 9 clusters were identified. Right panel displays the count and proportion of each cluster among the adaptive immune cells. (B) Dot plots depicting the list of canonical myeloid genes for annotation of myeloid cell UMAP. (C) Violin plot indicating the expression of *Tlr9* across identified myeloid clusters. (D) Violin plot indicating the expression of *Clec7a* across identified myeloid clusters. (E) Bar graph presenting the change in count and proportion of non-immune cells (low to no *Ptprc* expression) in the lungs across treatment and time. Cells were identified as follows: club cells (*Scgb1a1*), ciliated cells (*Rfx2*), Cycling AT2 (*Pdgfra*, *Top2a*, *Mki67*), Activated fibroblasts (*Col1a1*, *Col1a2*,

Serpinf2), Endothelial cells (*Lyve1*, *Pecam1*, *Cldn5*), AT2 cells (*Muc1*, *Sftpb*, *Sftpc*), lymphatic endothelial cells (*Pecam1*, *Thy1*, *Ccl21a*), and fibroblasts (*Col1a1*, *Col1a2*, *Sparc*, *Dcn*).

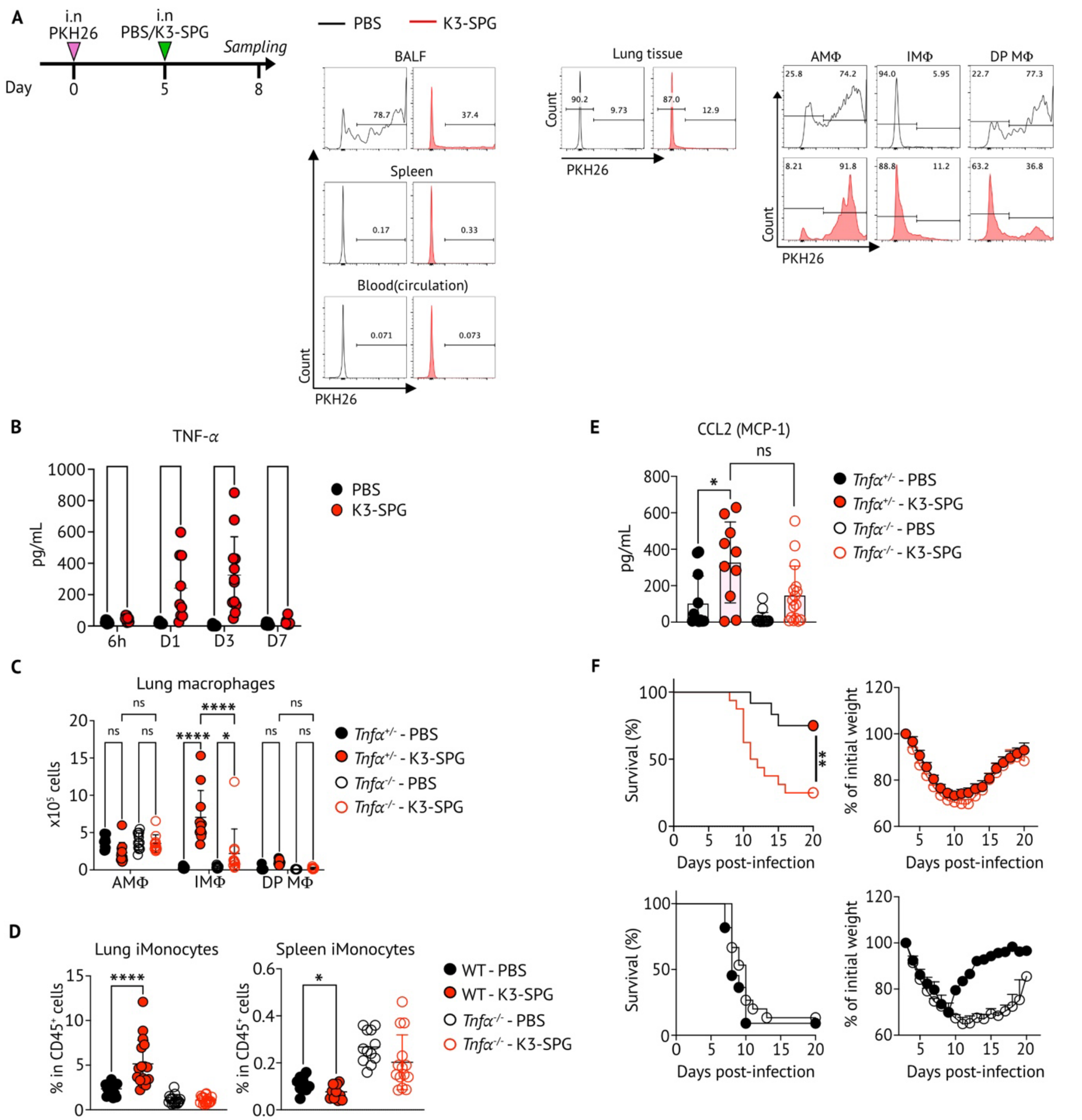

Fig. S4. Macrophage induction is dependent of TNF signaling.

(A) Intranasal PKH26 labelling does not leak into circulation or the spleen. Flow cytometry histograms showing PKH26 signal in the BAL fluid, lung tissue, blood, and spleen. Right panel demonstrates the proportion of $PKH26^{+}$ and $PKh26^{-}$ macrophage subsets in the lungs. Plots are representative of three independent experiments, with n=3 for each independent experiment. (B) Measurement of TNF-α in BAL fluid from day 1-, day 2-, and day3- pre-treated mice. (C) *Tnfα*$^{-/-}$ mice were treated with PBS or K3-SPG 3 days prior to sampling. The number of macrophage subsets in K3-SPG-treated *Tnfα*$^{+/-}$ and *Tnfα*$^{-/-}$ mice. Data is pooled from three independent experiments, with n≥3 for each independent experiment. (D) *Tnfα*$^{-/-}$ mice were treated with PBS or K3-SPG 3 days prior to sampling. The change of inflammatory monocytes in the lung and spleen, between WT or *Tnfα*$^{-/-}$ mice. Data is pooled from three independent experiments, with

n≥3 for each independent experiment. (E) Measurement of CCL2 (MCP-1) in BAL fluid from day 3 pre-treated *Tnfα* $^{+/-}$ and *Tnfα* $^{-/-}$ mice. Data is pooled from at least three independent experiments, with total N=10-16 mice per treatment group. (F) PR8 challenge using K3-SPG-treated *Tnfα*$^{+/-}$ and *Tnfα*$^{-/-}$ mice. Data is pooled from three independent experiments, with n=6-8 for each independent experiment. Two-way ANOVA with Tukey-corrected multiple comparisons, * = $p \leq 0.05$, **** = $p < 0.0001$ (C), Mann-Whitney test, * = $p \leq 0.05$, **** = $p < 0.0001$ [(D) and (E)], Survival comparison conducted with Kaplan-Maier log-rank (Mantel-Cox) test, * = $p < 0.05$, ** = $p < 0.01$ (F).

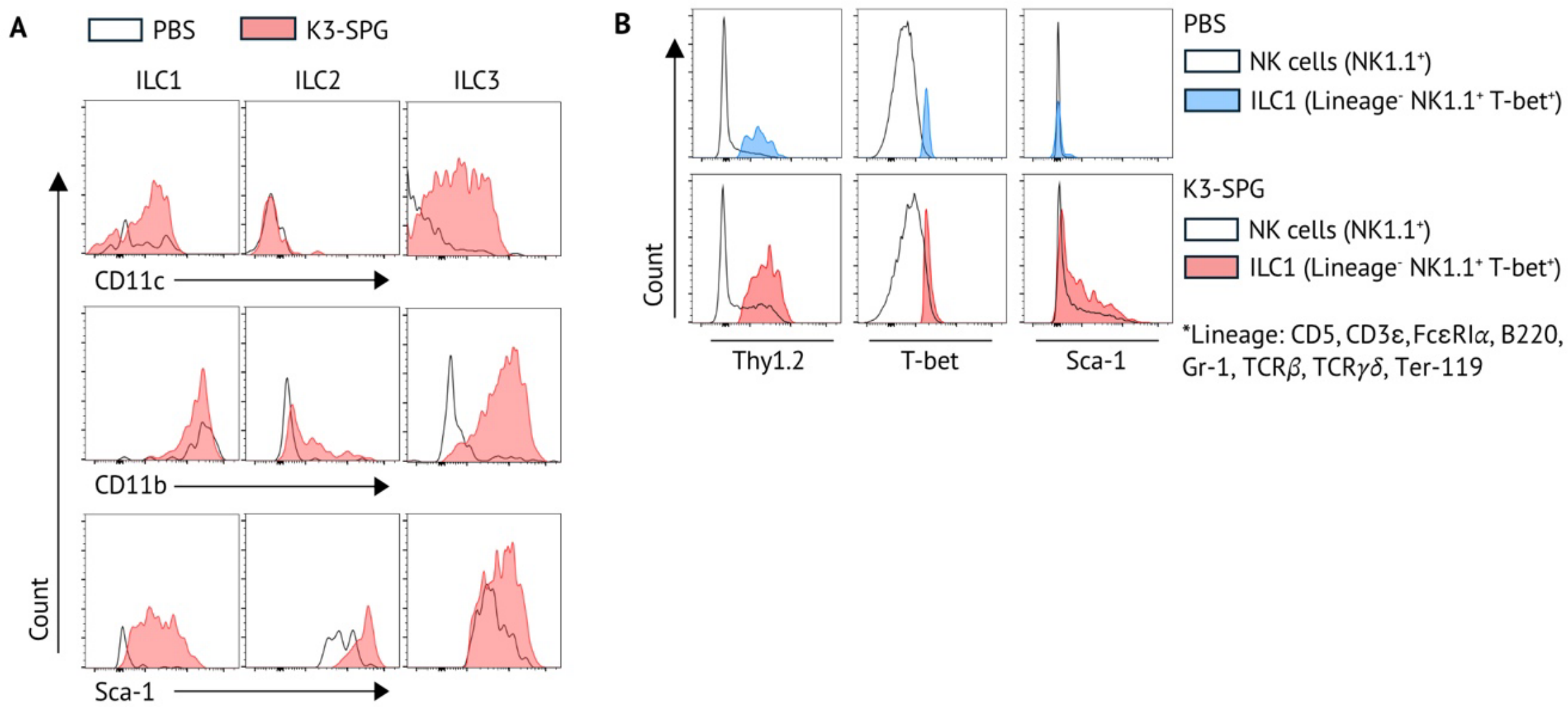

Fig. S5. ILCs sustain epigenetic modifications and confer long-term protection.
(A) Flow cytometry histograms showing gain of non-canonical surface marker expression by ILC1, ILC2, and ILC3 in day 3 pre-treated PBS- and K3-SPG-treated mice. (B) Distinguishing conventional NK cells and ILC1 based on Thy1.2, Sca-1, and T-bet expression.

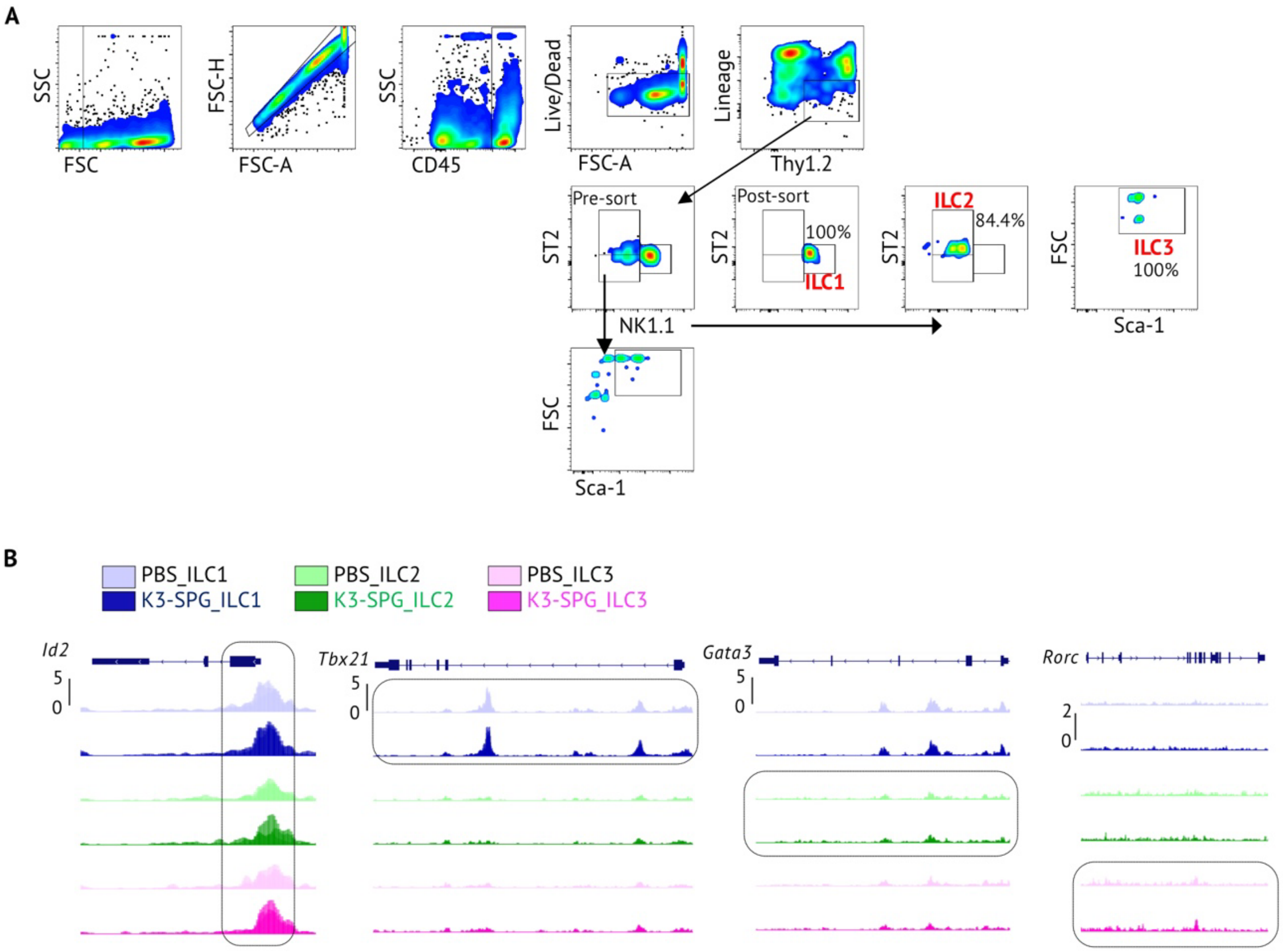

C

Motif	Status	Sequence	P-value	% of targets	% of background	Enriched in	Name
	De novo	ACTTCCYCTTTT	1e-209	51.78	19.36	K3-SPG_ILC2/3	SpiB(ETS)/OCILY3-SPIB-ChIP-Seq(GSE56857)/Homer(0.933)
	De novo	NDMTGASTCA	1e-42	32.46	18.85	K3-SPG_ILC2/3	Fra1(bZIP)/BT549-Fra1-ChIP-Seq(GSE46166)/Homer(0.970)
	De novo	CATATGAC	1e-17	40.89	31.19	K3-SPG_ILC2	MITF(bHLH)/MastCells-MITF-ChIP-Seq(GSE48085)/Homer (0.816)

Fig. S6. ILCs sustain epigenetic modifications and confer long-term protection.
(A) Sort gating strategy of ILCs without transcription factor staining for ATAC-sequencing. (B) IGV peaks demonstrating chromatin regions for ILC signature transcription factors in K3-SPG-treated ILCs. (C) Homer motif enrichment analysis was performed for ILC2 and ILC3, with sufficient number of DARs for motif analysis. Table depicts three motifs selected after correlation with genome peaks by IGV visualization.

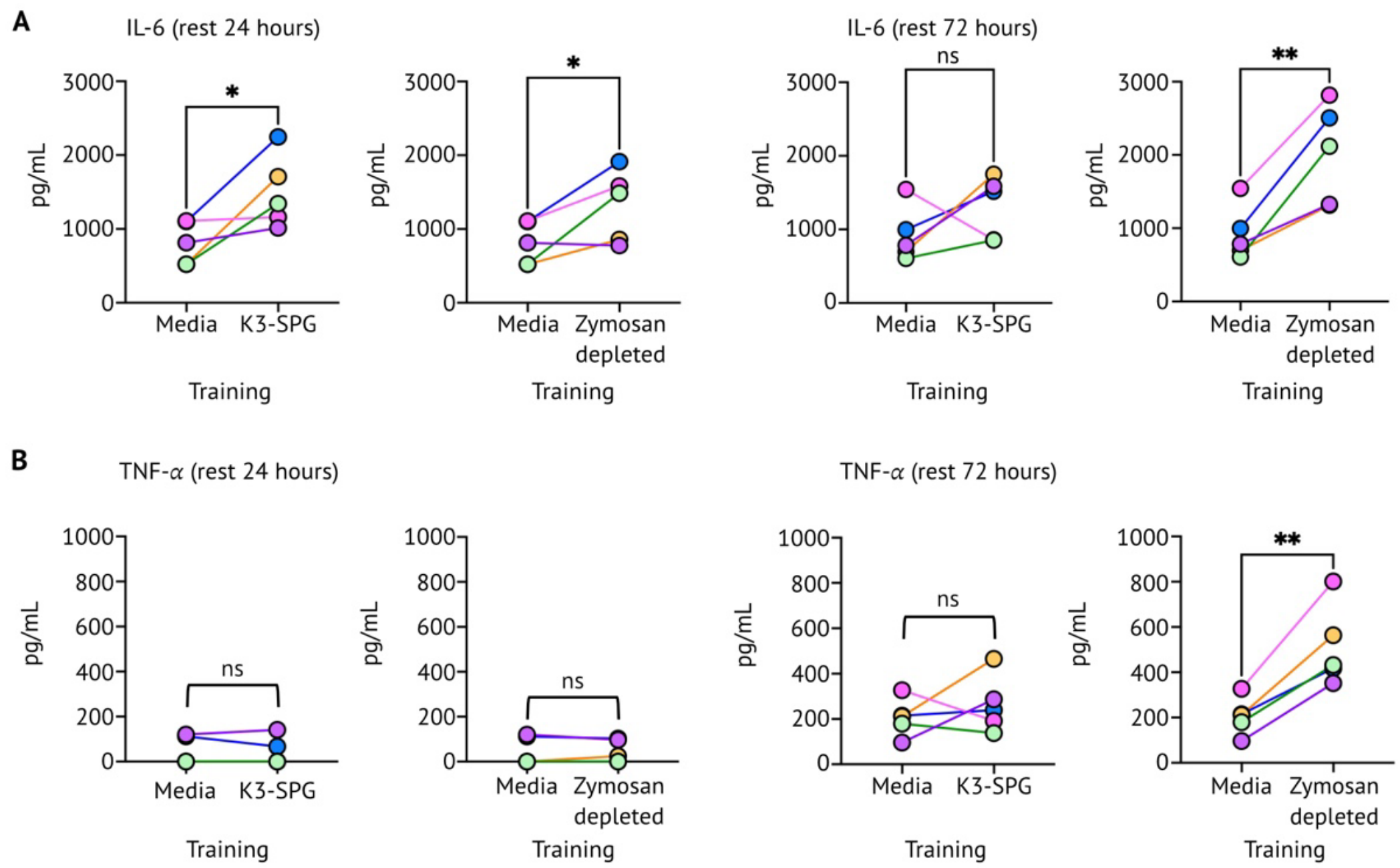

Fig. S7. K3-SPG trains human circulating leukocytes.
Leukocytes from five donors were stimulated with K3-SPG or zymosan depleted. After the initial 24 hour stimulation, adjuvants were washed and cells were rested in media for either 24 hour (left) or 72 hours (right). Cytokine induction was measured by ELISA. (A) IL-6 production in culture supernatant of leukocytes after LPS challenge. (B) TNF-α production in culture supernatant of leukocytes after LPS challenge. Statistical analysis was performed using a two-tailed paired t-test, * = $p < 0.05$, ** = $p < 0.01$.

Table S1. Enriched Homer motifs in ILC2.
List of all confirmed positive enriched *de novo* Homer motifs in ILC2 isolated from day 21 pre-treated mice.

Rank	Motif	P-value	% of targets	% of background	Best match/Details
1	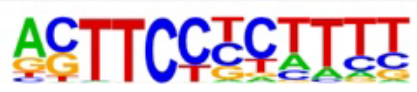	1e-209	51.78	19.36	SpiB(ETS)/OCILY3-SPIB-ChIP-Seq (GSE56857)/Homer(0.933)
2	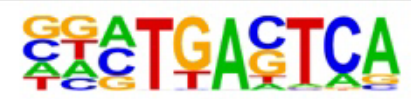	1e-42	32.46	18.85	Fra1(bZIP)/BT549-Fra1-ChIP-Seq (GSE46166)/Homer(0.970)
3	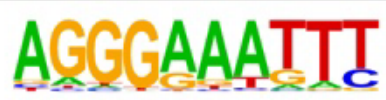	1e-20	9.96	4.68	RELB/MA1117.1/Jaspar(0.755)
4	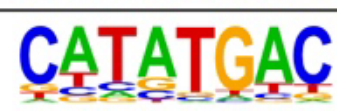	1e-17	40.89	31.19	MITF(bHLH)/MastCells-MITF-ChIP-Seq(GSE48085)/Homer(0.816)
5	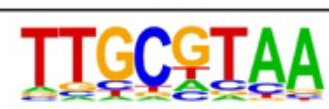	1e-17	13.96	7.90	CEBPB/MA0466.2/Jaspar(0.917)
6	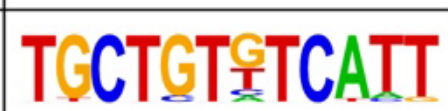	1e-14	1.04	0.08	Nfe2l2/MA0150.2/Jaspar(0.810)
7	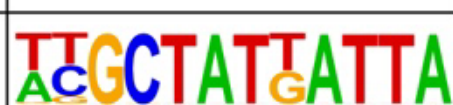	1e-13	0.49	0.01	MEF2B/MA0660.1/Jaspar(0.732)
8	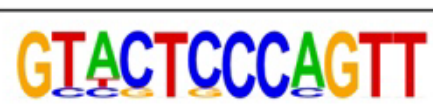	1e-13	0.55	0.01	Znf263(Zf)/K562-Znf263-ChIP-Seq (GSE31477)/Homer(0.671)
9	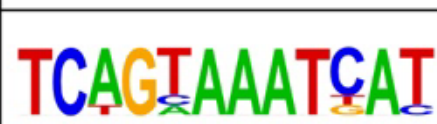	1e-12	0.49	0.01	EWS:ERG-fusion(ETS)/CADO_ES1-EWS:ERG-ChIP-Seq(SRA014231)/Homer(0.682)

Table S2. Enriched Homer motifs in ILC3.
List of top 10 confirmed positive enriched *de novo* Homer motifs in ILC3 isolated from day 21 pre-treated mice.

Rank	Motif	P-value	% of targets	% of background	Best match/Details
1	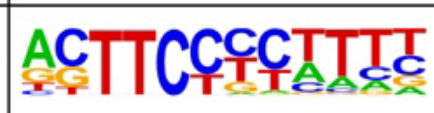	1e-611	42.47	13.68	SpiB(ETS)/OCILY3-SPIB-ChIP-Seq (GSE56857)/Homer(0.936)
2	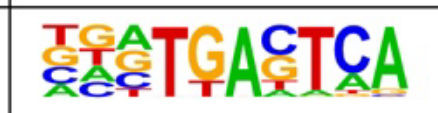	1e-180	30.8	15.51	Fra1(bZIP)/BT549-Fra1-ChIP-Seq (GSE46166)/Homer(0.979)
3	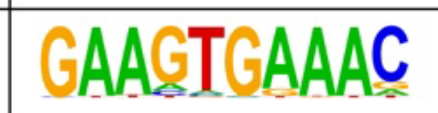	1e-107	6.83	1.77	PU.1-IRF(ETS:IRF)/Bcell-PU.1-ChIP-Seq(GSE21512)/Homer(0.944)
4	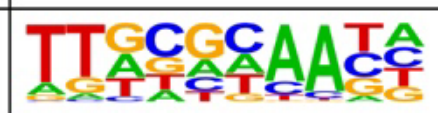	1e-70	14.67	7.67	CEBPE/MA0837.1/Jaspar(0.925)
5	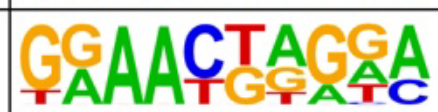	1e-51	7.30	3.19	IRF8(IRF)/BMDM-IRF8-ChIP-Seq (GSE77884)/Homer(0.644)
6	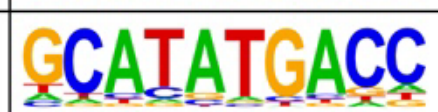	1e-22	6.51	3.79	NEUROG1/MA0623.2/Jaspar(0.738)
7	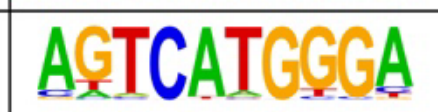	1e-16	2.36	1.04	USF1/MA0093.3/Jaspar(0.766)
8	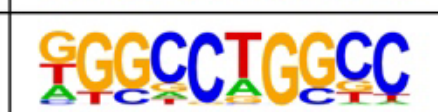	1e-16	14.37	10.81	ZNF711(Zf)/SHSY5Y-ZNF711-ChIP-Seq(GSE20673)/Homer(0.711)
9	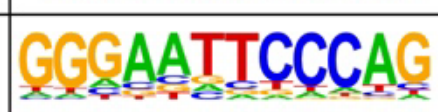	1e-15	8.17	5.55	NFkB-p65(RHD)/GM12787-p65-ChIP-Seq(GSE19485)/Homer(0.796)
10	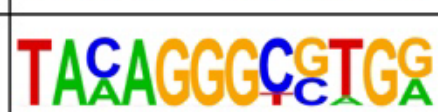	1e-13	0.23	0.01	Klf12/MA0742.1/Jaspar(0.738)
11	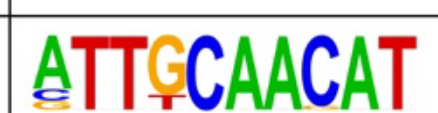	1e-13	0.55	0.10	CEBP:AP1(bZIP)/ThioMac-CEBPb-ChIP-Seq(GSE21512)/Homer(0.907)
12	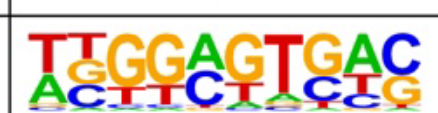	1e-13	2.03	0.92	Srebp1a(bHLH)/HepG2-Srebp1a-ChIP-Seq(GSE31477)/Homer(0.695)
13	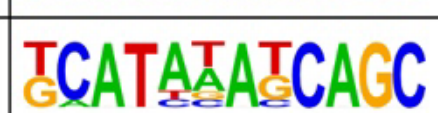	1e-13	0.23	0.01	PBX2/MA1113.2/Jaspar(0.737)
14	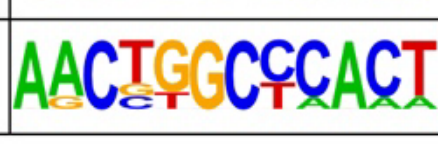	1e-12	0.26	0.02	MYB(HTH)/ERMYB-Myb-ChIPSeq (GSE22095)/Homer(0.587)
